# 7-Methoxyflavanone Alleviates LPS-Induced Acute Lung Injury by Suppressing TLR4/NF-κB p65 and ROS/Txnip/NLRP3 Signaling

**DOI:** 10.3390/biology14091170

**Published:** 2025-09-02

**Authors:** Kongyan Wang, Huiyu Hu, Zaibin Xu, Yan Chen, Yi Qiu, Yingjie Hu, Jiawen Huang, Zhuohui Luo

**Affiliations:** 1 Hainan Pharmaceutical Research and Development Science Park, Hainan Medical University, Haikou 571199, China; kywang2023@163.com (K.W.); 18672138207@163.com (H.H.); abinabab@163.com (Z.X.); cy052627@163.com (Y.C.); qiuyi_0927@163.com (Y.Q.); 2 Science and Technology Innovation Center, Guangzhou University of Chinese Medicine, Guangzhou 510405, China; yingjiehu@gzucm.edu.cn

**Keywords:** 7-Methoxyflavanone, acute lung injury, TLR4/NF-κB p65 signaling, ROS, Txnip/NLRP3 signaling

## Abstract

Background: Acute lung injury (ALI) is a serious respiratory condition. The natural compound 7-Methoxyflavanone (7MF) has a broad spectrum of anti-inflammatory and antioxidant properties. However, its pharmacological effects and underlying mechanisms in alleviating ALI remain poorly understood. Methods: An in vitro LPS-induced RAW264.7 macrophage inflammatory injury assay and an in vivo lipopolysaccharide (LPS)-induced ALI assay in mice were conducted. Results: In vitro experiments showed that 7MF significantly reduced levels of IL1β, IL6, and TNF-α; decreased the expression of COX2 and iNOS, as well as TLR4 and MyD88; suppressed the phosphorylation and degradation of IκBα; and blocked the entry of NF-κB p65 into the nucleus, thereby inhibiting NF-κB signaling. Meanwhile, 7MF also decreased ROS levels; prevented the dissociation of Txnip from Trx-1; and suppressed NLRP3, Caspase-1, Cleaved Caspase-1 p10, NEK7, Caspase-8, Cleaved Caspase-8, IL18, GSDMD, and GSDMD N-terminal expression, and thus inhibited NLRP3 signaling. MCC950, a specific inhibitor of NLRP3, significantly enhanced the pharmacological inhibition of NLRP3 by 7MF. Notably, similar results were confirmed in LPS-induced ALI experiments in mice. Conclusions: The compound 7MF effectively alleviated LPS-induced ALI by suppressing TLR4/NF-κB p65 and ROS/Txnip/NLRP3 signaling pathways. Our findings provide scientific evidence for drug development and treatment of ALI.

## Simple Summary

Acute lung injury (ALI) is a serious respiratory condition with complex causes and a lack of effective drugs or treatment methods. It has a mortality rate of up to 40%, posing a significant threat to patients’ health. The natural plant compound 7-Methoxyflavanone (7MF) has anti-inflammatory and antioxidant properties. However, its pharmacological effects on ALI remain unclear. In this study, researchers performed both in vitro and in vivo experiments to explore the molecular pharmacological mechanism of 7MF in alleviating ALI. The results further confirmed 7MF’s excellent anti-inflammatory activity effects and found that it ameliorates LPS-induced ALI by inhibiting TLR4/NF-κB p65 signaling-mediated inflammatory cascade response and ROS/Txnip/NLRP3 signaling-mediated cellular pyroptosis. Additionally, MCC950 enhanced 7MF’s pharmacological inhibition on NLRP3. These findings offer new insights and a scientific foundation for the clinical treatment of ALI.

## 1. Introduction

Acute lung injury (ALI), a serious respiratory condition, is a pro-inflammatory immune response triggered by recognizing pathogens in lung tissue during infection, resulting in diffuse interstitial alveolar edema and acute hypoxic respiratory failure [1,2]. It is also caused by a pro-inflammatory immune response initiated by the recognition of pathogens in lung tissue during microbial infection [3]. The pathogenesis of ALI is complex. Studies have demonstrated that ALI results in a significant influx of neutrophils into the lungs, which release pro-inflammatory cytokines that damage the lung epithelium and endothelium, impair gas exchange, and contribute to pulmonary edema [4]. In fact, the pathogenesis of ALI involves multiple signaling pathways, such as NF-κB-, MAPK-, JAK/STAT-, AMPK/SIRT3-, Nrf2/HO-1-, PI3K/Akt-, apoptosis-, pyroptosis-, and autophagy-related pathways [5]. Due to the complexity of ALI’s pathogenesis and the absence of effective drugs or treatments with minimal side effects, the mortality rate for ALI patients ranges from 30% to 40% [6]. Thus, developing drugs to alleviate lung tissue inflammation and damage is crucial.

Nuclear factor-kappa B (NF-κB) signaling, a well-known inflammatory regulatory pathway, is essential in the biological processes of ALI and pulmonary fibrosis [7]. Lipopolysaccharide (LPS), a component of Gram-negative bacteria’s cell walls, can trigger a rapid and intense inflammatory response that damages the normal function of immune cells, subsequently recruiting inflammatory cells into the lungs, and resulting in ALI [8,9]. When cells are unstimulated, IκBs retain inactive NF-κB dimers in the cytoplasm, which do not affect downstream gene transcription. However, when stimulation occurs via LPS, IκB becomes phosphorylated, which leads to NF-κB activation and its migration into the nucleus, thereby regulating the expression of inflammation-related genes and mediators [10].

It has long been confirmed that LPS-stimulated reactive oxygen species (ROS) production induces the dissociation of thioredoxin-interacting protein (Txnip) and thioredoxin-1 (Trx-1), which contributes to the interaction between Txnip and NLRP3, thereby activating the NLRP3 inflammasome [11]. NLRP3 inflammasome, a crucial multiprotein complex within the innate immune system, accelerates the release of IL1β and IL18, leading to lung inflammation and playing a critical role in the progression of ALI [12]. IL1β and IL18 are important pro-inflammatory factors during ALI. IL-1β enhances the recruitment of inflammatory cells and stimulates the release of significant inflammatory factors by binding to the IL-1 receptor on the cell surface and increasing the expression of adhesion factors [13]. IL18 induces inflammation by increasing intercellular cell adhesion molecule-1 (ICAM1) expression, nitric oxide synthesis, and chemokine production [14]. Hence, pharmacological inhibition of NF and NLRP3 signaling could attenuate the inflammatory response and cellular damage, exerting an ameliorative and protective effect against ALI.

Natural compounds derived from traditional Chinese medicine (TCM) have inspired drug development. Many natural products have demonstrated promising potential for treating ALI [15,16]. Our previous studies indicated that 7-Methoxyflavanone (7MF) inhibited LPS-stimulated TLR4/MyD88/MAPK signaling and activated the transcription of Nrf2-mediated antioxidant protein NQO-1, thereby exerting anti-neuroinflammatory effects in BV2 cells [17], considering that ALI represents an excessive inflammatory response in the lungs. Thus, based on LPS-stimulated ALI in mice and LPS-stimulated acute inflammatory injury experiments in RAW264.7 macrophages, this study aims to investigate the therapeutic effects and potential pharmacological mechanisms of the natural compound 7MF on ALI and to provide scientific evidence for the treatment of ALI.

## 2. Materials and Methods

### 2.1. Chemicals and Reagents

The compound 7-Methoxyflavone (7MF), with a purity exceeding 98.0%, was sourced from ACMEC Biochemical (Shanghai, China). LPS was obtained from Sigma-Aldrich (Shanghai, China). MCC950 was acquired from MCE (Shanghai, China). The antibodies for TLR4 (Cat No. 19811-1-AP) and MyD88 (Cat No. 67969-1-Ig) were obtained from Proteintech (Proteintech Group, Inc., Rosemont, IL, USA). The antibodies for IL6 (Cat No. DF6087), Phospho-IκBα (S32/S36) (Cat No. AF2002), IκBα (Cat No. AF5002), COX2 (Cat No. AF7003), iNOS (Cat No. AF0199), CD68 (Cat No. DF7518), MCP-1 (Cat No. DF7577), ICAM1 (Cat No. AF6088), VCAM1 (Cat No. DF6082), Phospho-NF-κB p65 (S536) (Cat No. AF2006), Cleaved Caspase-1 p10 (Cat No. AF4022), and Caspase-8 (Cat No. AF6442) were purchased from Affinity (Affinity Biosciences, Beijing, China). The antibody for TNF-α (Cat No. PY19810) was supplied by Abmart (Shanghai, China). The antibodies for NF-κB p65 (Cat No. YT3108), NLRP3 (Cat No. YT5382), ASC (Cat No. YT0365), Caspase-1 (Cat No. YT5743), IL1β (Cat No. YT5201), NEK7 (Cat No. YT3034), IL18 (Cat No. YN1926), GSDMD N-terminal (Cat No. YT7991), Cleaved Caspase-8 (Cat No. YC0011), and β-actin (Cat No. YM8343 and YM3028) were obtained from ImmunoWay (ImmunoWay, Plano, TX, USA).

### 2.2. Cell Culture and Cell Viability

RAW264.7 cells were cultured in Dulbecco’s Modified Eagle’s Medium (DMEM) containing 10% fetal bovine serum (FBS). The culture environment was kept at 37 °C with 5% CO_2_. For cell viability, cells were inoculated into a 96-well plate at a density of 2 × 10^4^ cells per well and incubated 24 h, then treated with 0, 2.5, 5, 10, 20, 40, 80, 160, and 320 μM of 7MF for an additional 24 h. The CCK-8 kit (Kumamoto, Japan) assessed cell viability following the manufacturer’s instructions. For subsequent cellular experiments, cells were cultured for 24 h prior to 7MF treatment, then stimulated with LPS (0.5 μg/mL) for 24 h. Cell supernatant or cells were subsequently collected for further analysis.

### 2.3. Measurement of NO

RAW264.7 cells were treated with 7MF at 20, 40, and 80 μM, followed by LPS stimulation for 24 h. The supernatant was collected to detect the nitrogen oxide (NO) (Cat No. S0021S) (Beyotime Biotechnology, Shanghai, China) level in accordance with the manufacturer’s instructions.

### 2.4. Animal Experiment

Male C57BL/6 mice, 8 weeks old, were purchased from the Guangdong Medical Laboratory Animal Center, weighing approximately 18–22 g, kept in an SPF environment with a 12 h light–dark cycle, and provided with a normal diet and drinking water. After a one-week adaptation period, 24 mice were randomly assigned to the control group (Con), LPS group (LPS), LPS + 7MF 40 mg/kg group, and LPS + 7MF 80 mg/kg group, with 6 mice per group. Except for the Con and LPS groups, the 7MF group received 40 mg/kg and 80 mg/kg of 7MF via oral gavage for 1 week, respectively. Subsequently, all groups except for the Con group were injected intraperitoneally with LPS (5 mg/kg) and monitored for 24 h. The mice were then euthanized, and bronchoalveolar lavage fluid (BALF), serum, and lung tissues were collected. All the experiments were approved by the Ethics Committee of Guangzhou University of Traditional Chinese Medicine (NO.20240322006).

### 2.5. Hematoxylin and Eosin Staining

Lung tissues were fixed in 4% paraformaldehyde for 48 h, dehydrated, embedded in paraffin, and cut into 5 μm-thick slices, followed by staining with hematoxylin and eosin (H&E) solution (Biosharp, Hefei, China). Images were captured using an electronic digital scanner (KONFOONG BIOINFORMATION TECH CO., LTD, Ningbo, China). Data are available in the Supplementary Materials.

### 2.6. Giemsa Staining

The obtained BALF was centrifuged to collect the supernatant, and the precipitated cells were then resuspended with PBS and stained with Wright–Giemsa (Cat No. C0131). Images were captured using a digital microscope scanner.

### 2.7. Enzyme-Linked Immunosorbent Assay

The levels of TNF-α (Cat No. 88-7324-86), IL-6 (Cat No. 88-7064-86), and IL1β (Cat No. 88-7013A-86) in the supernatants from RAW264.7 cells were measured using uncoated enzyme-linked immunosorbent assay (ELISA) kits (Invitrogen, Carlsbad, CA, USA) in accordance with the manufacturer’s instructions.

### 2.8. Lung Tissues Wet/Dry (W/D) Ratio

The collected lung tissues were immediately weighed wet and transferred to an oven set at 80 °C for 48 h to dry. The dry weight was measured, and the W/D ratio was calculated to evaluate pulmonary edema.

### 2.9. Measurement of MDA and SOD

Serum levels of malondialdehyde (MDA) (Cat No. A003-1-2) and superoxide dismutase (SOD) (Cat No. A001-3-2) were measured using commercial assay kits (Nanjing Jiancheng Bioengineering Institute, Nanjing, China) in accordance with the manufacturer’s instructions.

### 2.10. Immunofluorescence

RAW264.7 cells were fixed in 4% paraformaldehyde solution, incubated with 0.2% Triton X-100, and then blocked with 1% BSA. For lung tissues, slices were dewaxed and hydrated, incubated with 3% H_2_O_2_, and then blocked with PBS containing 1% BSA. Samples were incubated at 4 °C overnight with specific primary antibodies (dilution ratio 1:200–500), followed by incubation with the secondary antibody at room temperature for 1 h (dilution ratio 1:200–1000). Finally, the nucleus was re-stained with DAPI. Images were captured with an electronic digital scanner (Konfoong Bioinformation Tech Co., Ltd., Ningbo, China). Data are available in the Supplementary Materials.

### 2.11. Immunoblotting

Total proteins from RAW264.7 cells and lung tissues were extracted using RIPA buffer containing phenylmethylsulfonyl fluoride (PMSF) and phosphatase inhibitors. The BCA kit was used to measure protein concentrations. Subsequently, equal amounts of cellular proteins were separated using sodium dodecyl sulfate (SDS)-polyacrylamide gel electrophoresis (PAGE) and then transferred to methanol-activated polyvinylidene difluoride (PVDF) membranes. The membranes were blocked with a rapid sealing fluid and incubated at 4 °C overnight with specific primary antibodies (dilution ratio 1:1000–2000), followed by incubation with the secondary antibody at room temperature for 1 h (1: 10,000). Finally, bands were captured using an ECL reagent with the Molecular Imager^®^ System (Bio-Rad, Hercules, CA, USA) or an Amersham ImageQuant™ 800 System (Cytiva, Shanghai, China). ImageJ 1.54p (NIH, Bethesda, MD, USA, https://imagej.net/ij/) was used for quantification. The original data are available in the Supplementary Materials.

### 2.12. qPCR Analysis

Total mRNA from RAW264.7 cells or lung tissues was extracted using TRIzol reagent (Invitrogen™, ThermoFisher, Waltham, MA, USA) and reverse transcribed into cDNA. qPCR analysis was carried out with TB Green™ Premix ExTaq™ (Takara Biomedical Technology Co., Ltd., Dalian, China) on the Bio-Rad CFX Connect Real-Time System (Shanghai, China). The amplification program included 1 cycle at 95 °C for 30 s, followed by 40 cycles at 95 °C for 5 s and 60 °C for 30 s. Melting curve analysis was performed immediately after amplification to confirm primer specificity. All protocols were performed in accordance with the manufacturer’s instructions. The relative mRNA levels of genes were normalized and calculated using the 2^−∆∆Ct^ method with β-actin as the internal reference. The list of primers used in this study is shown in Supplementary Table S1.

### 2.13. Statistical Analysis

All data were analyzed using GraphPad Prism 9.0 software (San Diego, CA, USA). One-way ANOVA followed by Dunnett’s multiple comparison test was conducted. Results are expressed as mean ± standard error of mean (SEM). *p* < 0.05 was deemed statistically significant.

## 3. Results

### 3.1. 7MF Inhibited LPS-Induced Inflammatory Response in RAW264.7 Cells

**Figure 1:**
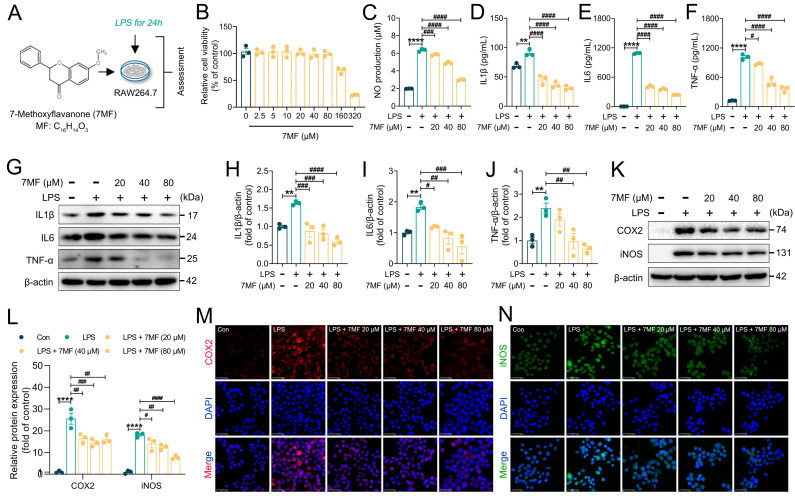
The 7MF inhibited LPS-induced inflammatory response in RAW264.7 cells. (**A**) Experimental process of in vitro experiments. (**B**) Cell viability. (**C**) NO production. (**D**–**F**) Levels of IL1β, IL6, and TNF-α in the culture supernatant of RAW264.7 cells. (**G**–**J**) Representative immunoblot and relative quantification of IL1β, IL6, and TNF-α in RAW264.7 cells. (**K**,**L**) Representative immunoblot and relative quantification of COX2 and iNOS in RAW264.7 cells. (**M**,**N**) Representative immunofluorescence images of COX2-positive and iNOS-positive in RAW264.7 cells. Scale bars: 25 μm. Data are expressed as mean ± SEM (n = 3). ** *p* < 0.01 and **** *p* < 0.0001 vs. the Con group. ^#^ *p* < 0.05, ^##^ *p* < 0.01, ^###^ *p* < 0.001, and ^####^ *p* < 0.0001 vs. the LPS group.

To reveal the molecular mechanism of 7MF alleviating ALI, LPS-induced acute inflammatory response in RAW264.7 cells was examined (Figure 1A). Cell viability revealed that 0 to 80 μM of 7MF did not have a significant impact on RAW264.7 cells (Figure 1B). Meanwhile, further experiments confirmed that 7MF did not inhibit LPS-dependent RAW264.7 cell proliferation (Supplementary Figure S1). Thus, based on activity results and a previous study [17], the concentrations of 7MF (20, 40, and 80 μM) were selected for subsequent experiments. As shown in Figure 1C–F, the levels of NO, IL1β, IL6, and TNF-α were significantly elevated after LPS stimulation, indicating severe inflammatory injury in the cells, whereas 7MF significantly reversed these inflammatory levels. Further immunoblotting analyses showed that 7MF significantly reduced LPS-stimulated protein levels of IL1β, IL6, and TNF-α (Figure 1G–J), demonstrating a strong ability to attenuate inflammatory injury. The original data are available in the Supplementary Materials. COX2 and iNOS are the main biomarkers of inflammatory response [18,19]. Interestingly, immunoblotting and immunofluorescence analysis revealed that 7MF also substantially suppressed the expression of COX2 and iNOS (Figure 1K–N), further confirming its excellent anti-inflammatory ability.

### 3.2. 7MF Suppressed TLR4/NF-κB p65 Signaling-Mediated Inflammatory Injury in RAW264.7 Cells

**Figure 2:**
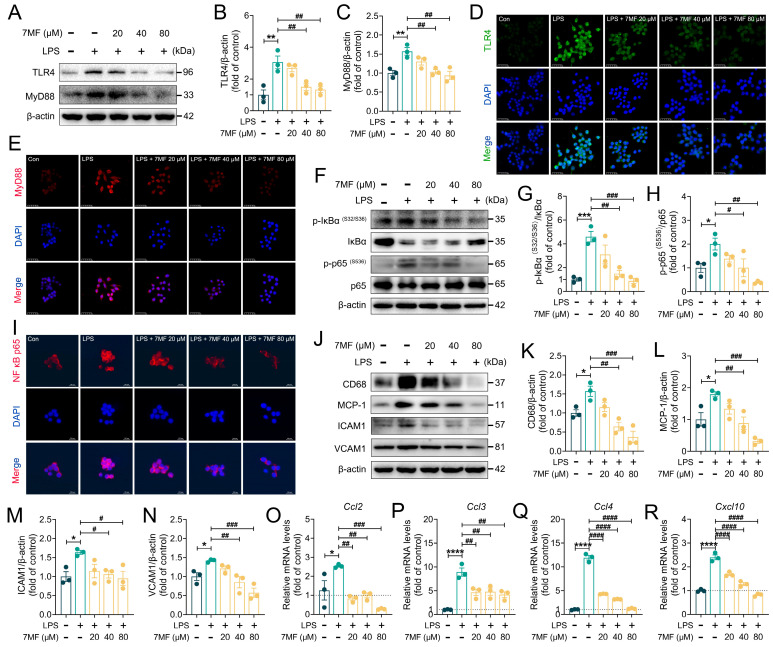
The 7MF suppressed TLR4/NF-κB p65 signaling-mediated inflammatory injury in RAW264.7 cells. (**A**–**C**) Representative immunoblot and relative quantification of TLR4 and MyD88 in RAW264.7 cells. (**D**,**E**) Representative immunofluorescence images of TLR4-positive and MyD88-positive in RAW264.7 cells. Scale bars: 25 μm. (**F**–**H**) Representative immunoblot and relative quantification of p-IκBα (S32/S36), IκBα, p-p65 (S536), and p65 in RAW264.7 cells. (**I**) Representative immunofluorescence images of NF-κB p65 in RAW264.7 cells. Scale bars: 100 μm. (**J**–**N**) Representative immunoblot and relative quantification of CD68, MCP-1, ICAM1, and VCAM1 in RAW264.7 cells. (**O**–**R**) mRNA levels of *Ccl2*, *Ccl3*, *Ccl4*, and *Cxcl10* in RAW264.7 cells. Data are presented as mean ± SEM (n = 3). * *p* < 0.05, ** *p* < 0.01, *** *p* < 0.001, and **** *p* < 0.0001 vs. the Con group. ^#^ *p* < 0.05, ^##^ *p* < 0.01, ^###^ *p* < 0.001, and ^####^ *p* < 0.0001 vs. the LPS group.

Toll-like receptor 4 (TLR4), an important receptor that responds to LPS stimulation, activates MyD88/NF-κB signaling-mediated inflammatory signaling [20,21,22]. In the present study, to examine the effect of 7MF on LPS-stimulated NF-κB signaling in RAW264.7 cells, immunoblotting and immunofluorescence analyses were used to assess the expression levels of TLR4, MyD88, Phospho-IκBα, IκBα, Phospho-NF-κB p65, and NF-κB p65. As shown in Figure 2A–I, 7MF markedly reduced the expression levels of TLR4, MyD88, Phospho-IκBα, and Phospho-NF-κB p65, inhibited the phosphorylation and degradation of IκBα, preventing the translocation of cytoplasmic NF-κB p65 into the nucleus, thereby blocking the activation of NF-κB signaling. Notably, in the classical NF-κB-signaling activation induced by LPS, TLR4 is an important target protein upstream of NF-κB signaling. To further explore the molecular pharmacological effects of 7MF on TLR4, we conducted molecular docking and molecular dynamics simulation analyses (Supplementary Materials). Interestingly, the results showed that 7MF has good affinity and low binding free energy with TLR4, suggesting that 7MF can stably bind to TLR4 and exert pharmacological activity (Supplementary Figure S2, Supplementary Table S2). These findings suggested that 7MF excellently modulated the inhibitory effect of NF-κB signaling. In addition, further experimental analysis confirmed this result. The 7MF markedly suppressed the expression of inflammation-related mediators CD68, MCP-1, ICAM1, and VCAM1 proteins (Figure 2J–N), as well as reduced the mRNA levels of chemokine *Ccl2*, *Ccl3*, *Ccl4*, and *Cxcl10* genes (Figure 2O–R), demonstrating that 7MF brilliantly inhibited TLR4/NF-κB p65 signaling to ameliorate cellular inflammatory damage.

### 3.3. 7MF Inhibited ROS/Txnip/NLRP3 Signaling-Mediated Pyroptosis in RAW264.7 Cells

**Figure 3:**
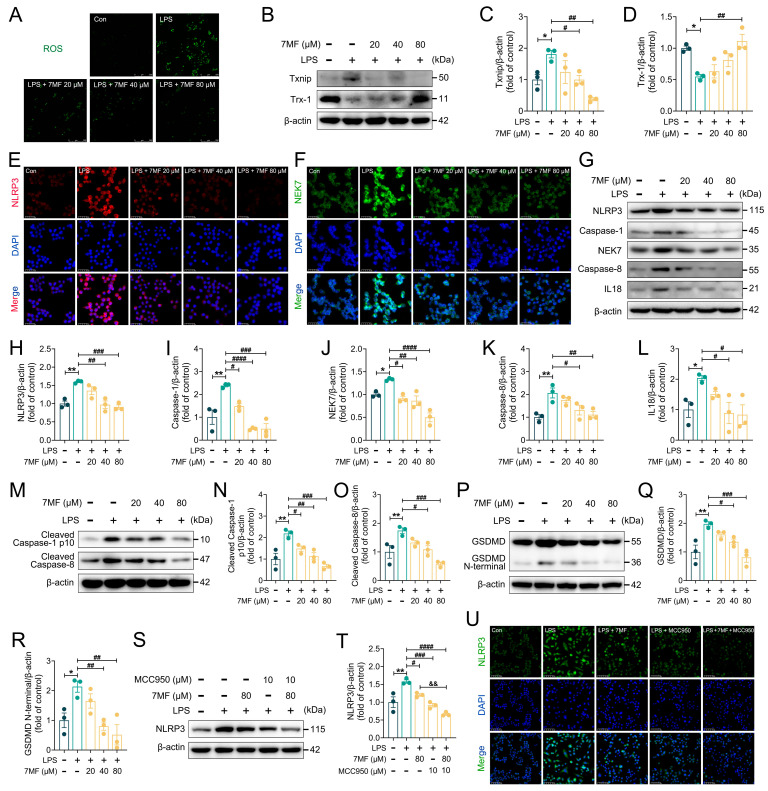
The 7MF inhibited ROS/Txnip/NLRP3 signaling-mediated pyroptosis in RAW264.7 cells. (**A**) ROS level in RAW264.7 cells. Scale bars: 250 μm. (**B**–**D**) Representative immunoblot and relative quantification of Txnip and Trx-1 in RAW264.7 cells. (**E**,**F**) Representative immunofluorescence images of NLRP3-positive, NEK7-positive in RAW264.7 cells. Scale bars: 25 μm. (**G**–**L**) Representative immunoblot and relative quantification of NLRP3, Caspase-1, NEK7, Caspase-8, and IL18 in RAW264.7 cells. (**M**–**O**) Representative immunoblot and relative quantification of Cleaved Caspase-1 p10 and Cleaved Caspase-8 in RAW264.7 cells. (**P**–**R**) Representative immunoblot and relative quantification of GSDMD and GSDMD N-terminal in RAW264.7 cells. (**S**,**T**) Representative immunoblot and relative quantification of NLRP3 in RAW264.7 cells after MCC950 intervention. (**U**) Representative immunofluorescence images of NLRP3-positive in RAW264.7 cells after MCC950 intervention. Scale bars: 50 μm. Data are presented as mean ± SEM (n = 3). * *p* < 0.05 and ** *p* < 0.01 vs. the Con group. ^#^ *p* < 0.05, ^##^ *p* < 0.01, ^###^ *p* < 0.001, and ^####^ *p* < 0.0001 vs. the LPS group. ^&&^ *p* < 0.01 vs. the 7MF group.

Existing studies demonstrated that an imbalance between ROS generation and clearance activates the NLRP3 inflammasome, resulting in varying degrees of tissue damage [23,24]. LPS, a potent activator of immune cells, has been shown to induce ROS production. Notably, the generated ROS triggers the dissociation of Txnip from Trx-1, leading to Txnip binding to NLRP3 and consequently activating inflammasomes, which stimulates IL1β and IL18 secretion and causes inflammatory cell pyroptosis [25]. In this study, 7MF substantially decreased LPS-stimulated ROS production (Figure 3A), inhibited Txnip expression, and increased Trx-1 expression (Figure 3B–D), exerting its ability to block NLRP3 inflammasome activation from upstream signaling. Unsurprisingly, further immunofluorescence analysis revealed that 7MF markedly suppressed the expression of NLRP3 and NEK7 (Figure 3E,F). Meanwhile, immunoblotting confirmed the results that 7MF also significantly reduced the expression of NLRP3, Caspase-1, NEK7, Caspase-8, IL18, Cleaved Caspase-1 p10, Cleaved Caspase-8, GSDMD, and GSDMD N-terminal proteins (Figure 3G–R). Furthermore, MCC950, an NLRP3-specific antagonist, dramatically enhanced the pharmacological inhibition of LPS-stimulated NLRP3 by 7MF (Figure 3S–U). Collectively, all these findings indicated that 7MF attenuated cellular inflammatory injury by suppressing ROS/Txnip/NLRP3 signaling-mediated pyroptosis.

### 3.4. 7MF Reduced Infiltration and Exudation of Pro-Inflammatory Cells in ALI Mice

**Figure 4:**
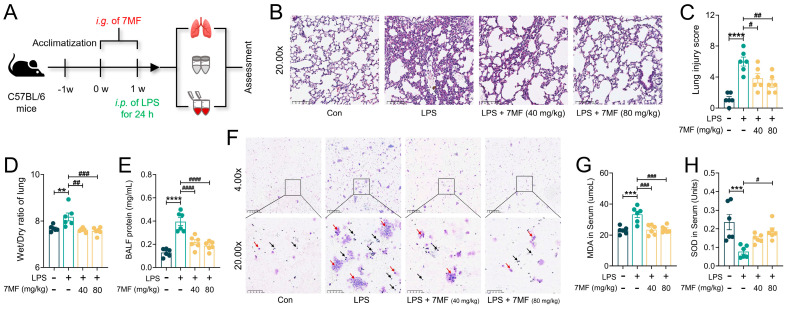
The 7MF reduced infiltration and exudation of pro-inflammatory cells in ALI mice. (**A**) Study design of in vivo experiment. (**B**) Representative images of H&E staining in lung tissues. Original magnification, 20.00×. (**C**) Lung injury score (n = 6). (**D**) Wet/Dry ratio of the lung (n = 6). (**E**) BALF protein level (n = 6). (**F**) Giemsa staining in BALF. Original magnification, 4.00× and 20.00×. Neutrophils in light purple-red (arrows: black) and macrophages in blue-purple (arrows: red). (**G**,**H**) MDA and SOD levels in serum (n = 6). Data are expressed as mean ± SEM. ** *p* < 0.01, *** *p* < 0.001 and **** *p* < 0.0001 vs. the Con group. ^#^ *p* < 0.05, ^##^ *p* < 0.01, ^###^ *p* < 0.001, and ^####^ *p* < 0.0001 vs. the LPS group.

In vitro experiments demonstrated that 7MF exhibited excellent inhibitory effects on LPS-induced inflammation in RAW264.7 cells. To further assess the molecular pharmacological mechanisms of 7MF in ameliorating cellular inflammatory injury to alleviate ALI, an LPS-induced mouse model of ALI was used for evaluation (Figure 4A). As shown in Figure 4B, the pathological changes in lung tissue induced by LPS stimulation were primarily characterized by increased inflammatory cell aggregation and alveolar hemorrhage. The original captured images are available in the Supplementary Materials. Based on Smith’s scoring system [26], 7MF markedly reduced LPS-stimulated lung injury (Figure 4C). However, 7MF significantly mitigated these severe pathological changes. Furthermore, 7MF also decreased the Wet/Dry ratio of the lung and reduced protein leakage (Figure 4D,E), thereby ameliorating LPS-induced pulmonary edema injury. Interestingly, Giemsa-staining results confirmed the effectiveness of 7MF in reducing the total number of inflammatory cells in BALF, such as neutrophils (light purple-red, arrows: black) and macrophages (blue-purple, arrows: red) (Figure 4F), suggesting that 7MF helps reduce the infiltration and exudation of pro-inflammatory cells in lung tissue. Further analysis revealed that 7MF also reduced MDA levels and increased SOD activity in serum (Figure 4G,H), ameliorating LPS-induced ALI by attenuating oxidative damage in tissue cells.

### 3.5. 7MF Alleviated ALI in Mice by Inhibiting Inflammatory Response

**Figure 5:**
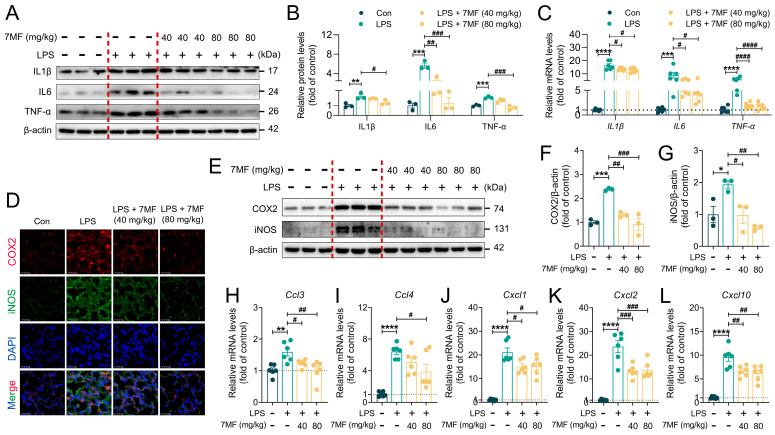
The 7MF alleviated ALI in mice by inhibiting the inflammatory response. (**A**,**B**) Representative immunoblot and relative quantification of IL1β, IL6, and TNF-α in lung tissues (n = 3). (**C**) mRNA levels of IL1β, IL6, and TNF-α genes in lung tissues (n = 6). (**D**) Representative immunofluorescence images of COX2-positive and iNOS-positive in lung tissues. Scale bars: 25 μm. (**E**–**G**) Representative immunoblot and relative quantification of COX2 and iNOS in lung tissues (n = 3). (**H**–**L**) mRNA levels of *Ccl3*, *Ccl4*, *Cxcl1*, *Cxcl2*, and *Cxcl10* in lung tissues (n = 6). Data are expressed as mean ± SEM. * *p* < 0.05, ** *p* < 0.01, *** *p* < 0.001, and **** *p* < 0.0001 vs. the Con group. ^#^ *p* < 0.05, ^##^ *p* < 0.01, ^###^ *p* < 0.001, and ^####^ *p* < 0.0001 vs. the LPS group.

Excessive inflammatory responses can lead to ALI by causing neutrophil accumulation in lung tissue, interstitial edema, and damage to alveolar epithelial cells [27]. To further explore how 7MF inhibited the inflammatory response, immunoblotting, immunofluorescence, and qPCR analyses were used for assessment. Consistent with the in vitro observations mentioned above, 7MF treatment decreased IL1β, IL6, and TNF-α expression dose-dependently at both the protein and mRNA levels (Figure 5A–C). COX2 and iNOS are key enzymes that contribute to the onset and progression of ALI during the inflammatory response [28]. As expected, LPS-stimulated mice showed a significant increase in COX2 and iNOS expression in lung tissue, while 7MF intervention reversed these effects (Figure 5D–G), suggesting a critical role for 7MF in suppressing the inflammatory response. The original images are available in the Supplementary Materials. Given that chemokines are essential signaling molecules that mediate the inflammatory response [29], in this study, LPS-induced chemokines such as *Ccl3*, *Ccl4*, *Cxcl1*, *Cxcl2*, and *Cxcl10* were significantly elevated. Interestingly, 7MF markedly reduced the mRNA levels of these genes in murine lung tissues (Figure 5H–L). Collectively, these results suggested that 7MF ameliorates LPS-induced ALI by reducing the inflammatory response.

### 3.6. 7MF Inhibited TLR4/NF-κB p65 Signaling-Mediated Inflammatory Injury in ALI Mice

**Figure 6:**
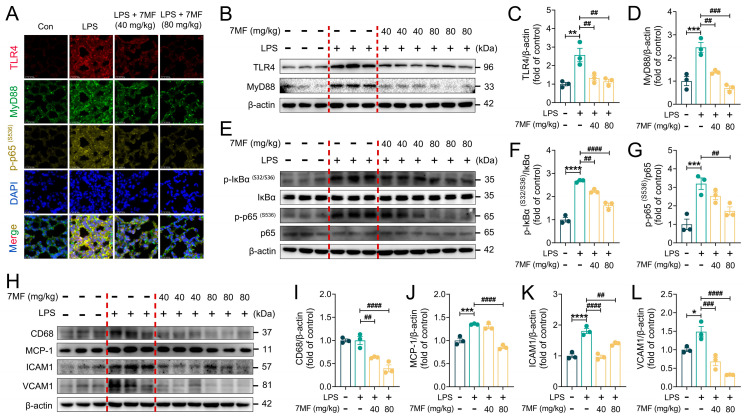
The 7MF inhibited TLR4/NF-κB p65 signaling-mediated inflammatory injury in ALI mice. (**A**) Representative immunofluorescence images of TLR4-positive, MyD88-positive, and p-p65 (S536)-positive in lung tissues. Scale bars: 25 μm. (**B**–**D**) Representative immunoblot and relative quantification of TLR4 and MyD88 in lung tissues (n = 3). (**E**–**G**) Representative immunoblot and relative quantification of p-IκBα (S32/S36), IκBα, p-p65 (S536), and p65 in lung tissues (n = 3). (**H**–**L**) Representative immunoblot and relative quantification of CD68, MCP-1, ICAM1, and VCAM1 in lung tissues (n = 3). Data are expressed as mean ± SEM. * *p* < 0.05, ** *p* < 0.01, *** *p* < 0.001, and **** *p* < 0.0001 vs. the Con group. ^##^ *p* < 0.01, ^###^ *p* < 0.001, and ^####^ *p* < 0.0001 vs. the LPS group.

To further explore how 7MF alleviates ALI, the effects of LPS-induced ALI on the TLR4/NF-κB signaling pathway were examined. Immunofluorescence staining results of lung tissue sections showed that LPS stimulation markedly elevated the expression levels of TLR4, MyD88, and Phospho-NF-κB p65, while 7MF treatment reversed the expression of these inflammatory mediators (Figure 6A). Further mechanistic studies demonstrated that 7MF markedly reduced TLR4, MyD88, Phospho-IκBα, and Phospho-NF-κB p65 expression (Figure 6B–G), as well as suppressed the expression of the downstream proteins CD68, MCP-1, ICAM1, and VCAM1 of NF-κB signaling in a dose-dependent manner (Figure 6H–L). These findings were consistent with the in vitro experiments described above, further confirming the excellent anti-inflammatory ability of 7MF in reducing LPS-induced ALI by suppressing TLR4/NF-κB p65 signaling-mediated inflammatory injury in ALI mice.

### 3.7. 7MF Suppressed Txnip/NLRP3 Signaling-Mediated Pyroptosis in ALI Mice

**Figure 7:**
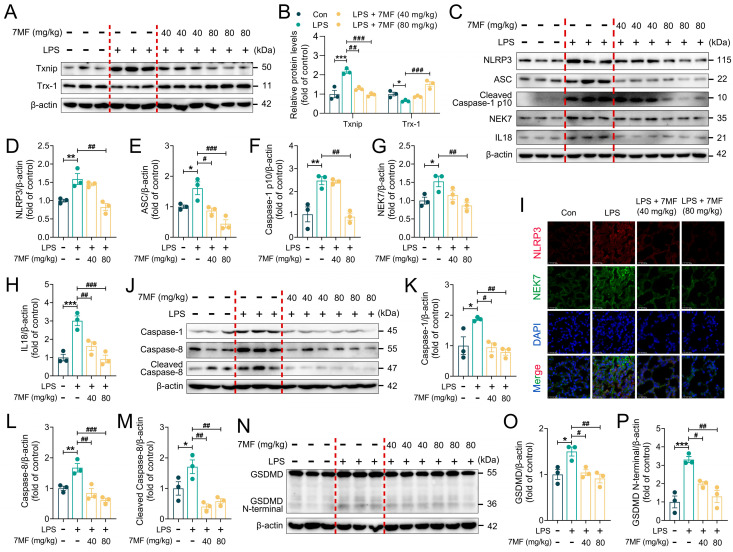
The 7MF suppressed Txnip/NLRP3 signaling-mediated pyroptosis in ALI mice. (**A**,**B**) Representative immunoblot and relative quantification of Txnip and Trx-1 in lung tissues (n = 3). (**C**–**H**) Representative immunoblot and relative quantification of NLRP3, ASC, Cleaved Caspase-1 p10, NEK7, and IL18 proteins in lung tissues (n = 3). (**I**) Representative immunofluorescence images of NLRP3-positive and NEK7-positive in lung tissues. Scale bars: 25 μm. (**J**–**M**) Representative immunoblot and relative quantification of Caspase-1, Caspase-8, and Cleaved Caspase-8 in lung tissues (n = 3). (**N**–**P**) Representative immunoblot and relative quantification of GSDMD and GSDMD N-terminal in lung tissues (n = 3). Data are presented as mean ± SEM. * *p* < 0.05, ** *p* < 0.01, and *** *p* < 0.001 vs. the Con group. ^#^ *p* < 0.05, ^##^ *p* < 0.01, and ^###^ *p* < 0.001 vs. the LPS group.

The results of in vitro experiments confirmed that ROS produced by mitochondrial metabolism could affect the expression of Txnip and Trx-1, thereby regulating the activation of the NLRP3 inflammasome and triggering the NLRP3/ASC/Caspase-1-mediated inflammatory cascade response. As expected, 7MF significantly decreased the expression of Txnip and increased the expression of Trx-1 (Figure 7A,B), which in turn prevented the dissociation of Txnip from Trx-1. Additionally, the immunoblotting results showed that 7MF also markedly suppressed the expression of NLRP3, ASC, Caspase-1 p10, NEK7, IL18, and GSDMD (Figure 7C–H). Further immunofluorescence analyses confirmed that 7MF reduced the expression of NLRP3 and NEK7 (Figure 7I). Moreover, given the significance of Caspase-1 activation, Cleaved Caspase-8, and GSDMD N-terminal in cellular pyroptosis, the expression levels of these proteins were evaluated through immunoblotting. As expected, 7MF significantly decreased the expression of Caspase-1, Cleaved Caspase-8, GSDMD, and GSDMD N-terminal (Figure 7J–P). Together, these findings suggested that 7MF alleviated LPS-induced ALI by inhibiting Txnip/NLRP3 signaling-mediated pyroptosis.

## 4. Discussion

ALI is a prevalent respiratory disease characterized by multiple and complex pathogenic factors [3]. Previous studies have demonstrated that the pathogenesis of ALI involves several signaling pathways, including NF-κB-, MAPK-, JAK/STAT-, AMPK/SIRT3-, Nrf2/HO-1-, PI3K/Akt-, apoptosis-, pyroptosis-, and autophagy-related pathways [5]. Excessive inflammatory response can aggravate the damage to the organism, thereby accelerating the onset and progression of ALI. Thus, early and effective interventions to control lung injury development are extremely important. LPS, a component of Gram-negative bacteria’s cell walls, has been extensively utilized to model inflammatory responses to evaluate the therapeutic effects of drugs [30]. Our previous studies have demonstrated that 7MF significantly inhibits LPS-stimulated TLR4/MyD88/MAPK signaling activation in BV2 cells, thereby exerting anti-neuroinflammatory effects [17]. This study aimed to investigate the protective effects of 7MF, a natural compound derived from TCM that exhibits both in vitro and in vivo therapeutic effects on LPS-induced ALI, while also revealing its potential pharmacological mechanisms.

LPS, a component of the outer membrane of Gram-negative bacterial cells, is an endotoxin that targets TLR4 on the surface of host cell membranes. TLR4, the main LPS receptor activated by LPS, initiates MyD88-dependent downstream signaling, promotes the phosphorylation and degradation of IκBα, allows the dimeric NF-κB p65 to translocate to the nucleus, and activates the transcription of target genes [10]. This process causes inflammatory cells to infiltrate and promotes the production of various inflammatory factors and mediators, including IL1β, IL6, TNF-α, COX2, and iNOS, which are crucial in ALI [8,9]. Indeed, during ALI, macrophages play a key role in the lung’s inflammatory response by releasing inflammatory factors (IL-1β, IL-6, and TNF-α), chemokines, and other molecules to recruit additional immune cells and participate in initiating and amplifying inflammation. RAW264.7 cells, a type of typical macrophage, are commonly used to simulate the behavior and function of macrophages during ALI to evaluate the molecular pharmacological mechanisms of drugs in resisting acute inflammatory responses.

In the current study of LPS-stimulated inflammatory injury in RAW264.7 cells and ALI in mice, 7MF significantly reduced pro-inflammatory cytokines levels such as IL1β, IL6, and TNF-α, and it possessed significant anti-inflammatory properties. NF-κB p50/p65, the most prevalent heterodimer in NF-κB signaling, becomes activated upon stimulation and enters the nucleus to modulate the release of pro-inflammatory cytokines, playing a crucial role in innate and adaptive immune, inflammatory, and stress responses [31]. Further pharmacological mechanism experiments revealed that 7MF significantly inhibited TLR4 expression on the cell membrane surface. This pharmacological activity was confirmed through molecular docking and molecular dynamics simulation analysis, thereby blocking upstream stimulation of signaling, inhibiting intracellular signaling, reducing MyD88 expression, decreasing the phosphorylation and degradation of IκBα, and preventing NF-κB p65 from entering the nucleus from the cytoplasm to regulate the transcription and expression of inflammatory factors and mediators, including biomarkers CD68, MCP-1, ICAM1, and VCAM1, suggesting the excellent inhibitory ability of 7MF to suppress LPS-stimulated inflammatory responses. Together, these findings confirmed that 7MF could alleviate ALI by ameliorating cellular inflammatory damage through blocking NF-κB signaling.

During the progression of ALI, the pathological changes are typically characterized by pulmonary edema, pulmonary and alveolar hemorrhage, and inflammatory cell infiltration [4]. Notably, LPS-stimulated overproduction of ROS can result in cellular oxidative stress, ultimately leading to severe pulmonary inflammation and exacerbating the onset and progression of ALI [32]. In this study, 7MF significantly decreased BALF protein concentration, indicating that it effectively inhibits protein leakage in lung tissue. Additionally, Giemsa staining showed that 7MF markedly reduced LPS-stimulated infiltration of inflammatory cells in the lung, such as neutrophils and macrophages. However, the analysis of BALF has some limitations, such as the absence of cell classification counts. These shortcomings will be the focus of our efforts in the next step. Additionally, MDA, one of the main products of lipid peroxidation, serves as a biomarker for oxidative stress. In contrast, SOD is one of the primary biological enzymes widely present in living organisms, which can effectively scavenge ROS and plays a crucial role in maintaining the organism’s oxidative balance [33]. In the current study, 7MF was found to significantly inhibit ROS production, lower MDA levels, and significantly enhance SOD activity, suggesting that 7MF may alleviate LPS-induced ALI via antioxidative stress.

It has long been established that Trx1 and Txnip play a crucial role in NLRP-3 inflammasome activation in a redox-dependent way [25]. Trx/Txnip, a redox-sensitive signaling complex, is a crucial part of the connection between redox regulation and disease pathogenesis. LPS-stimulated ROS cause the dissociation of Txnip from Trx-1 [11], prompting Txnip to bind to NLRP3, thereby inducing an inflammatory response by stimulating IL1β secretion through an NLRP3/ASC/Caspase-1 signaling-dependent pathway [34]. Caspase-8, an effector and regulator of NLRP3 inflammasome signaling, serves as a pro-apoptotic initiator and key IL-1β converting protease that activates NLRP3 inflammasome in the absence of Caspase-1. Conversely, when Caspase-1 is present, Caspase-8 positively regulates the NLRP3-dependent Caspase-1 signaling cascade, thus promoting IL-1β production and pyroptosis [35]. Additionally, NEK7 has been shown to be a crucial mediator of NLRP3 activation following potassium efflux [36]. Interestingly, in this current study, 7MF significantly reduced ROS accumulation and inhibited the dissociation of Txnip/Trx-1, thus preventing Txnip from binding to NLRP3, blocking the activation of NLRP3/ASC/Caspase-1 inflammasome, and reducing the expression of these essential biomarkers, thus inhibiting NLRP3 activation. MCC950, a specific NLRP3 inhibitor, significantly enhanced the suppressive effect of 7MF against NLRP3 when intervening in combination with 7MF. Of note, gasdermin D (GSDMD), a component of the gasdermin family, acts as a substrate for inflammatory Caspases-1. Notably, Caspase-1 specifically cleaves the linker between the amino-terminal gasdermin-N and the carboxy-terminal gasdermin-C domains in GSDMD, which plays a crucial role in the downstream effects of cellular pyroptosis [37,38]. In this study, further molecular mechanistic studies revealed that 7MF also inhibited the expression of GSDMD, thereby inhibiting GSDMD cleavage to release the N-terminal fragment, blocking GSDMD-N from initiating cellular pyroptosis by forming a pore on the cell membrane [39,40], suggesting that it may ameliorate ALI by inhibiting ROS/Txnip/NLRP3 signaling-mediated pyroptosis.

This study first demonstrated 7MF’s remarkable ability to inhibit inflammatory responses and cellular damage, providing a scientific basis for attenuating LPS-induced ALI. However, further and more in-depth research is still needed to elucidate its specific molecular pharmacologic mechanisms. Indeed, LPS-induced ALI involves immune responses from various cell types in lung tissue, such as alveolar macrophages, lung epithelial cells, and lung endothelial cells, emphasizing the roles and importance of cellular diversity [41,42]. The innate immune response of lung tissue acts as a vital defense against infections, reflecting the cellular functions within the lung [43,44]. Macrophages in lung tissue are diverse in type and are classified into alveolar macrophages, interstitial macrophages, perivascular macrophages, and inflammatory macrophages based on their origin or anatomical location in the lung. They play a crucial role in maintaining the homeostasis of lung tissue by regulating immune responses [45]. In this study, given the limitations of in vitro studies using RAW264.7 macrophages and the complexity of mechanisms involved in ALI pathogenesis, future research will consider macrophage cell lines closely associated with ALI, such as bone marrow-derived macrophages (BMDMs) [46], J774A.1 cells [47], or TPH-1 cells [48] to further explore the molecular pharmacology of 7MF in alleviating ALI. Meanwhile, considering the crucial role of communication and interaction between macrophages and lung epithelial cells in pulmonary inflammatory diseases [49], as well as advances in modern molecular pharmacology techniques (such as activity-based protein profiling, ABPP) [46,50], focusing on intercellular co-culture studies, activity-based probes (ABPs), proteomics methods to identify protein targets of active small molecules, and single-cell sequencing analysis [51] will help to further clarify the pathogenesis of ALI, offering new insights into the specific molecular pharmacology mechanisms of 7MF-targeted regulation in alleviating ALI.

## 5. Conclusions

**Figure 8:**
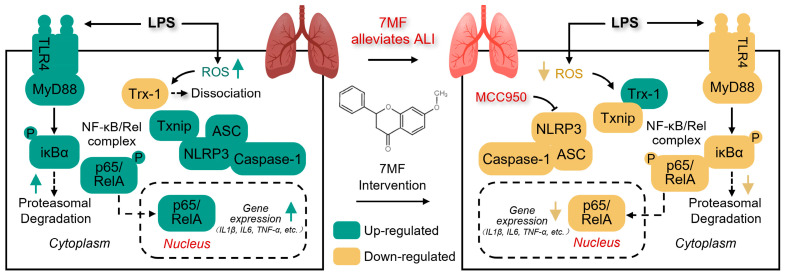
Diagram of how pharmacological mechanisms of 7MF alleviate LPS-induced ALI in this study.

In summary, the current study’s findings clearly demonstrate that 7MF alleviated LPS-induced ALI by inhibiting TLR4/NF-κB p65 signaling-mediated acute inflammatory response and ROS/Txnip/NLRP3 signaling-mediated pyroptosis (Figure 8). These results provide scientific evidence for preventing and treating ALI.

## Data Availability

The data presented in this study are available on request from the corresponding author due to privacy reasons.

## Supplementary Materials

The following supporting information can be downloaded at: https://www.mdpi.com/article/10.3390/biology14091170/s1.

## Abbreviations

The following abbreviations are used in this manuscript:

ALI	Acute lung injury
BALF	Bronchoalveolar lavage fluid
DMEM	Dulbecco’s modified Eagle’s medium
ELISA	Enzyme-linked immunosorbent assay
FBS	Fetal bovine serum
H&E	Hematoxylin and eosin
HRP	Horseradish peroxidase
ICAM1	Intercellular cell adhesion molecule-1
LPS	Lipopolysaccharide
MDA	Malondialdehyde
NF-κB	Nuclear factor-kappa B
NO	Nitrogen oxide
PAGE	Polyacrylamide gel electrophoresis
PMSF	Phenylmethylsulfonyl fluoride
ROS	Reactive oxygen species
SDS	Sodium dodecyl sulfate
SEM	Standard error of mean
SOD	Superoxide dismutase
TCM	Traditional Chinese medicine
TLR4	Toll-like receptor 4
Trx-1	Thioredoxin-1
Txnip	Thioredoxin-interacting protein
7MF	Methoxyflavanone
