# Supplementary material for: 7-Methoxyflavanone Alleviates LPS-Induced Acute Lung Injury by Suppressing TLR4/NF-κB p65 and ROS/Txnip/NLRP3 Signaling
Source: Biology (Basel). 2025 Sep 2;14(9):1170. doi: 10.3390/biology14091170 (PMC12467840; doi:10.3390/biology14091170)
Supplement: Supplementary file 1 [file biology-14-01170-s001.zip › biology-3787441-supplementary.pdf]

**Supplementary Material**

## 1. Methods

### *1.1. Molecular docking analysis*

The structure of compound 7MF was obtained from the PubChem database and then imported into Chem3D software for optimization and energy minimization using the MM2 module. The optimized structure was saved as an SDF format file to serve as the ligand molecule for molecular docking. The protein structure of TLR4 (PDB ID: 3VQ2) was obtained from the RCSB database (https://www.rcsb.org/) and processed using Schrodinger's Protein Preparation Wizard for energy minimization and geometric structure optimization. Molecular docking was performed using the Glide module in Schrodinger Maestro software. Finally, the interaction mode and docking scores between 7MF and TLR4 were analyzed to determine whether 7MF possesses any biological activity.

### *1.2. Molecular Dynamics Simulation*

Molecular dynamics simulations of 7MF and TLR4 were conducted using Gromacs 2020. Proteins were modeled with the AMBER99SB-ILDN force field, while small-molecule ligands were built using the sobtop program to generate topologies, with charge fitting done via RESP. The TIP3P explicit water model was used, with the minimum distance between protein atoms and the water box edge set to 1.0 nm. Sodium or chloride ions were added to neutralize the system charge based on docking results. Initially, the heavy atoms of the protein (and small molecules) were constrained, and 10,000 steps of energy minimization were performed on the water molecules. Then, the constraints were released, and another 10,000 steps of energy minimization were performed on the entire system. The system was gradually heated to 300 K over 50 ps; after heating, it was equilibrated for 50 ps under the NPT ensemble. Finally, a 100 ns molecular dynamics simulation was run under the NPT ensemble. Trajectory data were saved every 20 ps, and analysis was conducted using the trjconv module. Binding free energy calculations for 7MF and TLR4 were performed with gmxMMPBSA in Gromacs 2020.

## 2. Results

### *2.1. 7MF binds stably with TLR4*

The pharmacological effect of 7MF in significantly inhibiting the TLR4/NF-κB signaling pathway has been confirmed. Given the importance of TLR4 as an upstream regulator of NF-κB signaling, in this study, we used molecular docking and molecular dynamics simulations to further explore the molecular mechanism behind 7MF's inhibition of TLR4 activation. Interestingly, we found that 7MF exhibits a low binding energy (-6.474 kcal/mol, less than -5.0 kcal/mol) with TLR4 in the binding pocket, indicating that 7MF can stably bind to TLR4 to exert its pharmacological inhibitory effect (Supplementary Figure S2A).

To further clarify the interaction between 7MF and TLR4, we conducted 100 ns molecular dynamics simulations on the TLR4-7MF complex. As shown in Supplementary Figure S2B, 7MF forms approximately one hydrogen bond with amino acids in the TLR4 pocket (hydrogen bond occupancy rate of 36.8%), which is crucial for stabilizing the binding between the small molecule and the protein. RMSD is calculated as the sum of deviations in atomic positions between the conformation at a given time and the initial conformation, serving as a key indicator of system stability and molecular flexibility. As shown in Supplementary Figure S2C, the fluctuations in 7MF are significantly smaller than those in TLR4's RMSD, indicating that the compound can preserve its binding position on the protein. RMSF can be used to identify the regions of conformational change in each amino acid along the protein chain during the simulation. A higher RMSF value indicates greater conformational fluctuation in the amino acid and more flexible movement of the residue. As shown in Supplementary Figure S2D, most of the amino acid conformational changes in the complex formed by the interaction between TLR4 and 7MF remain within a relatively stable range. To analyze the relative compactness and stability induced by binding at the primary and secondary sites in the TLR4-7MF complex, we measured the target protein's radius of gyration (Rg). Rg assesses the compactness of the TLR4 structure. We found that the Rg of the TLR4 protein increases flexibility and conformational space during the initial binding phase, creating conditions for subsequent compaction and conformational stabilization (Supplementary Figure S2E). Additionally, the solvent-accessible surface area (SASA) begins to decrease after kinetic adjustment, which benefits the stability of the complex (Supplementary Figure S2F). Notably, binding free energy is a key tool for analyzing changes in ligand binding modes by measuring the thermodynamic properties of ligands. A negative binding free energy (ΔGbinding energy) indicates system stability, while a positive value suggests

instability (Supplementary Table S2). In this study, van der Waals forces are especially important in stabilizing small molecules, followed by electrostatic interactions. The binding free energy of 7MF with the TLR4 protein is -12.26 ± 1.33 kcal/mol. Hydrogen bonds between the compound and the protein pocket contribute significantly to stability through electrostatic interactions (-11.06 ± 0.46 kcal/mol); van der Waals forces also play a crucial role (-20.59 ± 0.01 kcal/mol), showing the compound can stay stably in the protein pocket and has strong van der Waals interactions with nearby residues. Additionally, a decomposition analysis of the binding free energy reveals that the amino acids HIS: 158, SER: 182, TYR: 183, SER: 210, LEU: 211, and PHE: 262 are key to stabilizing the small molecule (Supplementary Figure S2G). In summary, these results demonstrate that 7MF has a strong affinity for the TLR4 protein, forming a stable complex that likely inhibits its activity.

**Supplementary Figure Legend**

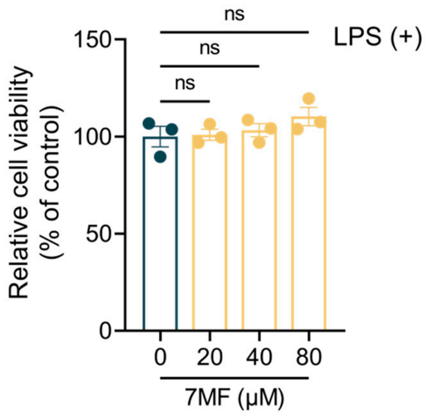


**Supplementary Figure S1.** 7MF did not inhibit LPS-dependent RAW264.7 cells proliferation. NS: no significance.

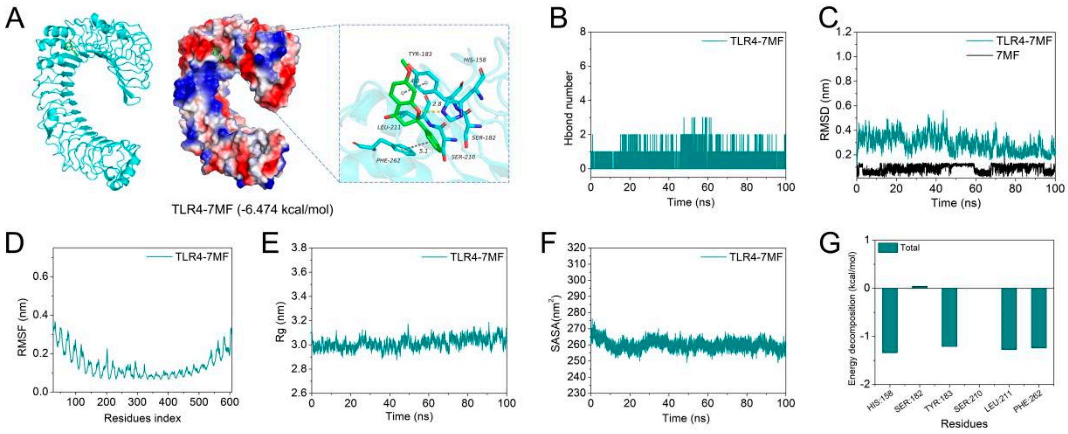


**Supplementary Figure S2.** 7MF binds stably with TLR4. (A) The binding mode of TLR4 with 7MF (The 3D structure of the complex. The electrostatic surface of a protein. The detailed binding mode of the complex. The backbone of the protein was rendered in a tube and colored green. Compound is rendered in green. (B) The hydrogen bond number of 7MF with TLR4. (C) The RMSD of 7MF with TLR4. (D) The RMSF of 7MF with TLR4. (E) The Rg of 7MF with TLR4. (F) The SASA of 7MF with TLR4. (G) The total energy decomposition of the binding free energy for TLR4-7MF.

**Supplementary Table S1.** Primers used in qPCR analysis.

| Gene | Forward Primer (5' → 3') | Reward Primer (5' → 3') |
|---|---|---|
| *Ccl2* | TTAAAAACCTGGATCGGAACCAA | GCATTAGCTTCAGATTTACGGGT |
| *Ccl3* | TTCTCTGTACCATGACACTCTGC | CGTGGAATCTTCCGGCTGTAG |
| *Ccl4* | TTCCTGCTGTTTCTCTTACACCT | CTGTCTGCCTCTTTTGGTCAG |
| *Cxcl1* | CTGGGATTCACCTCAAGAACATC | CAGGGTCAAGGCAAGCCTC |
| *Cxcl2* | CCAACCACCAGGCTACAGG | GCGTCACACTCAAGCTCTG |
| *Cxcl10* | CCAAGTGCTGCCGTCATTTTC | GGCTCGCAGGGATGATTTCAA |
| *IL1β* | CCGTGGACCTTCCAGGATGA | GGGAACGTCACACACCAGCA |
| *IL6* | TAGTCCTTCCTACCCCAATTTCC | TTGGTCCTTAGCCACTCCTTC |
| *TNF-α* | AGCCCCCAGTCTGTATCCTT | CTCCCTTTGCAGAACTCAGG |
| *β-actin* | GGCTGTATTCCCCTCCATCG | CCAGTTGGTAACAATGCCATGT |

**Supplementary Table S2.** The binding energy by MMPBSA (kcal/mol)

| Type | TLR4-7MF |
|---|---|
| $E_{VDW}$ | -20.59 ± -0.01 |
| $E_{ELE}$ | -11.06 ± -0.46 |
| $E_{GB}$ | 21.82 ± -1.25 |
| $E_{SA}$ | -2.43 ± -0.02 |
| $G_{Binding\ energy}$ | -12.26 ± -1.33 |

$E_{VDW}$: van der Waals energy

$E_{ELE}$: eletrostatic energy

$E_{GB}$: polar contribution to solvation

$E_{SA}$: non-polar contribution to solvation

# Original Scans of Immunoblots

## Fig. 1G

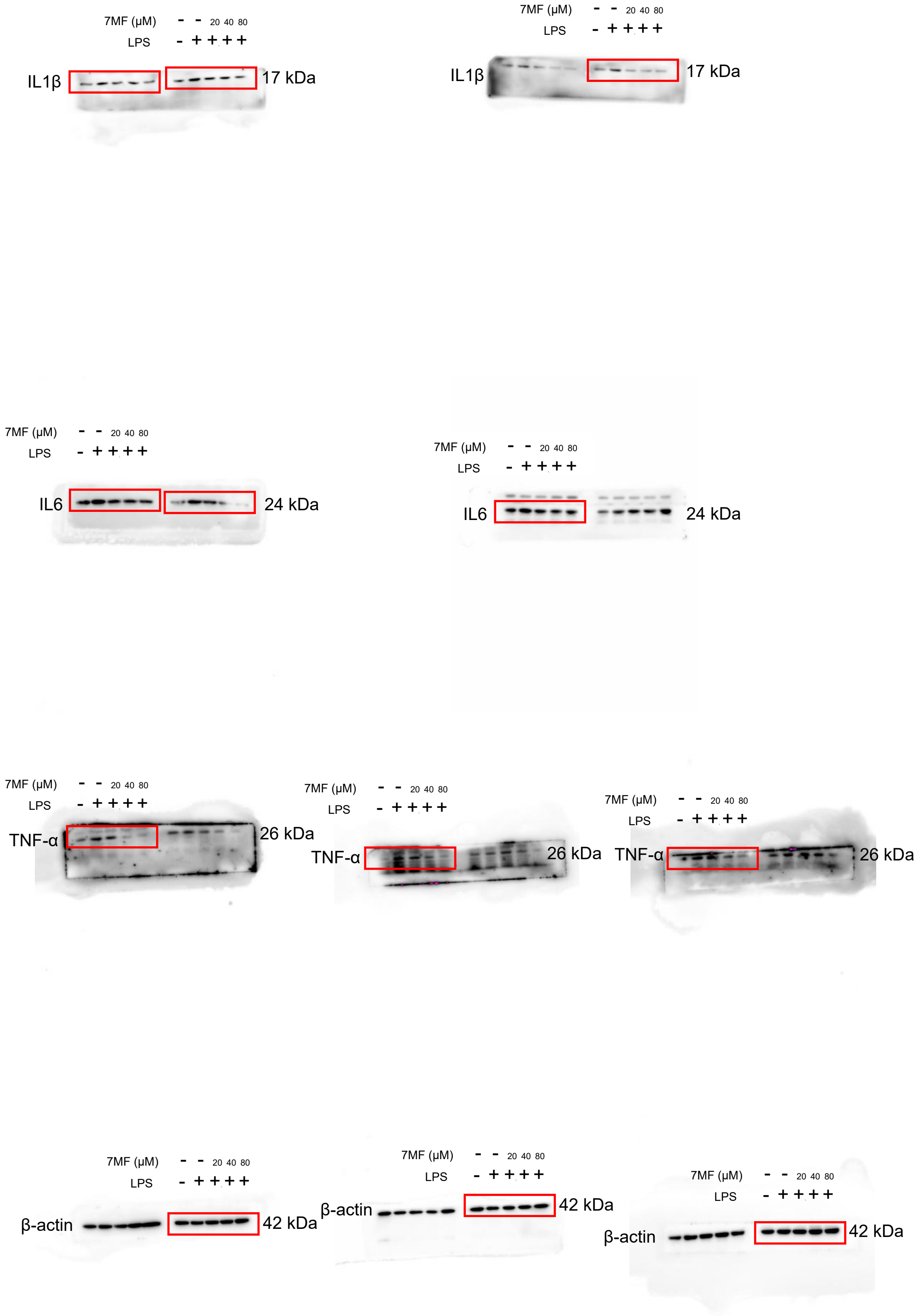

# Fig. 1K

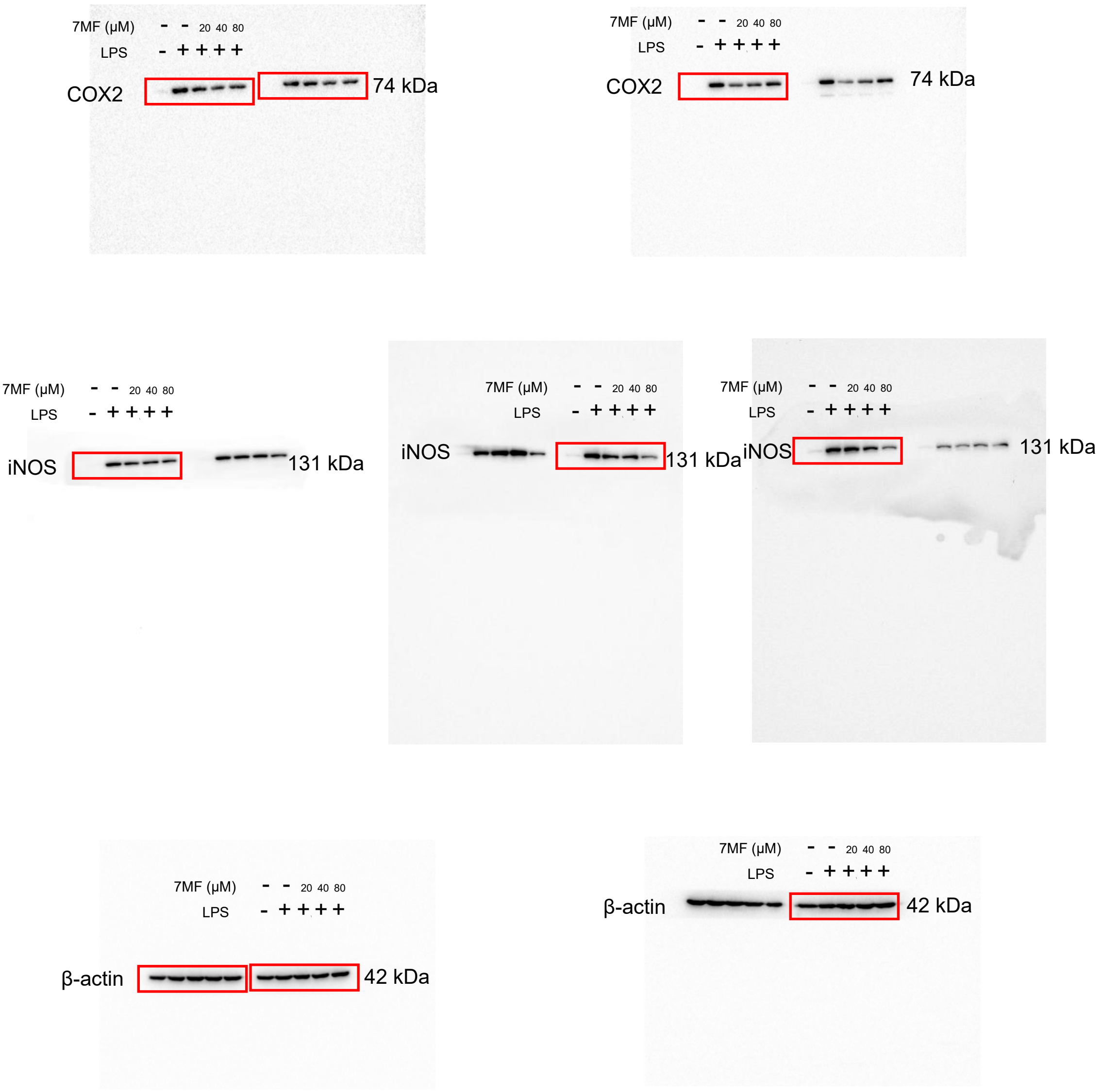
7MF (μM) - - 20 40 80
LPS - + + + +
COX2
74 kDa
7MF (μM) - - 20 40 80
LPS - + + + +
COX2
74 kDa
7MF (μM) - - 20 40 80
LPS - + + + +
iNOS
131 kDa
7MF (μM) - - 20 40 80
LPS - + + + +
iNOS
131 kDa
7MF (μM) - - 20 40 80
LPS - + + + +
iNOS
131 kDa
7MF (μM) - - 20 40 80
LPS - + + + +
β-actin
42 kDa
7MF (μM) - - 20 40 80
LPS - + + + +
β-actin
42 kDa

**Fig. 2A**

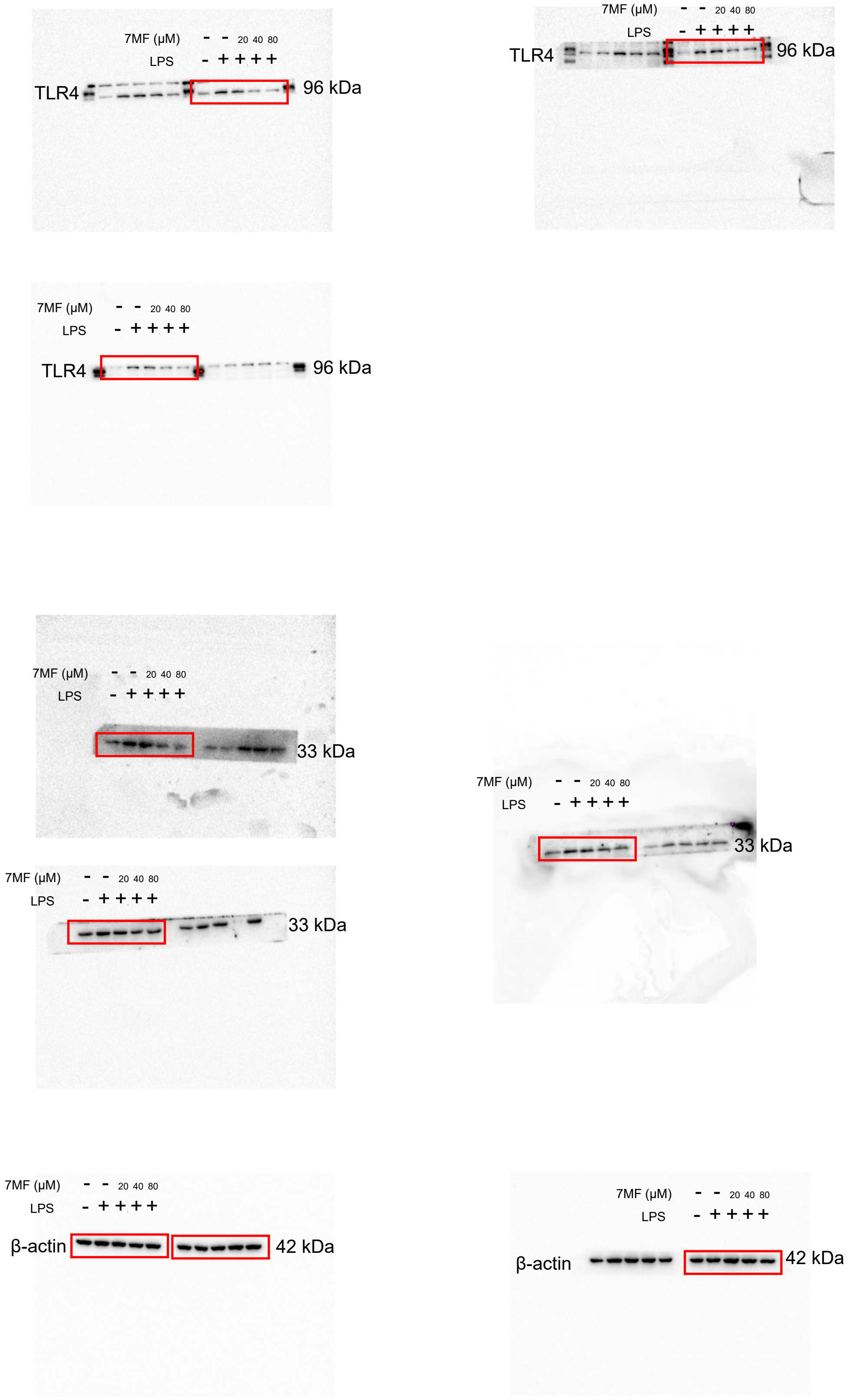
7MF (μM) - - 20 40 80
LPS - + + + +
TLR4
96 kDa
7MF (μM) - - 20 40 80
LPS - + + + +
TLR4
96 kDa
7MF (μM) - - 20 40 80
LPS - + + + +
TLR4
96 kDa
7MF (μM) - - 20 40 80
LPS - + + + +
33 kDa
7MF (μM) - - 20 40 80
LPS - + + + +
33 kDa
7MF (μM) - - 20 40 80
LPS - + + + +
33 kDa
7MF (μM) - - 20 40 80
LPS - + + + +
β-actin
42 kDa
7MF (μM) - - 20 40 80
LPS - + + + +
β-actin
42 kDa

**Fig. 2F**

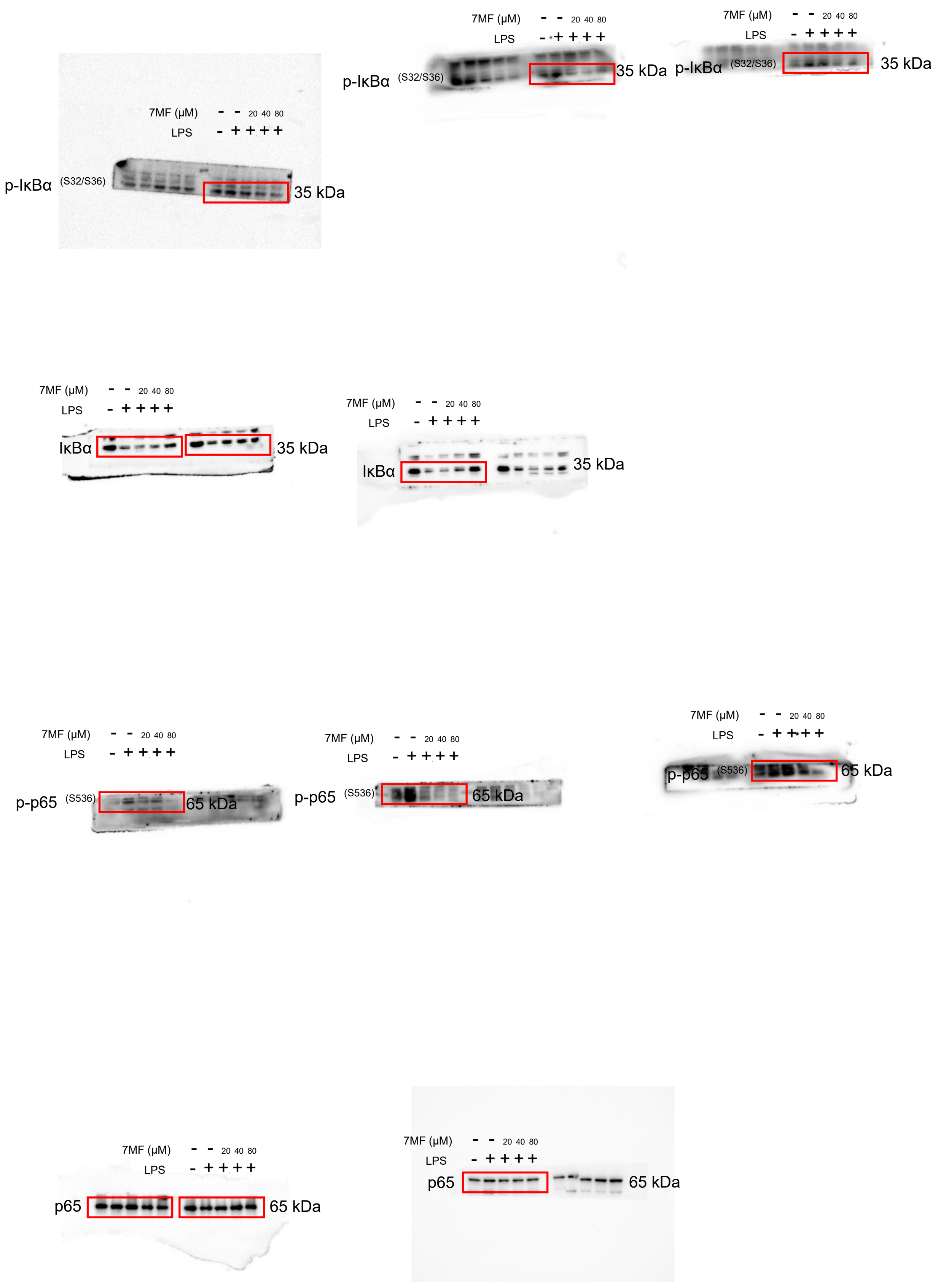
7MF (μM) - - 20 40 80
LPS - + + + +
p-IκBα (S32/S36)
35 kDa
7MF (μM) - - 20 40 80
LPS - + + + +
p-IκBα (S32/S36)
35 kDa
7MF (μM) - - 20 40 80
LPS - + + + +
p-IκBα (S32/S36)
35 kDa
7MF (μM) - - 20 40 80
LPS - + + + +
IκBα
35 kDa
7MF (μM) - - 20 40 80
LPS - + + + +
IκBα
35 kDa
7MF (μM) - - 20 40 80
LPS - + + + +
p-p65 (S536)
65 kDa
7MF (μM) - - 20 40 80
LPS - + + + +
p-p65 (S536)
65 kDa
7MF (μM) - - 20 40 80
LPS - + + + +
p-p65 (S536)
65 kDa
7MF (μM) - - 20 40 80
LPS - + + + +
p65
65 kDa
7MF (μM) - - 20 40 80
LPS - + + + +
p65
65 kDa

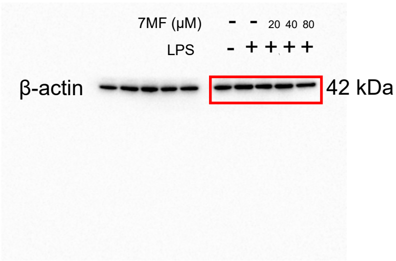
7MF (μM)
- - 20 40 80
LPS
- + + + +
β-actin
42 kDa

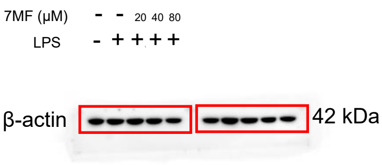
7MF (μM)
- - 20 40 80
LPS
- + + + +
β-actin
42 kDa

Fig. 2J

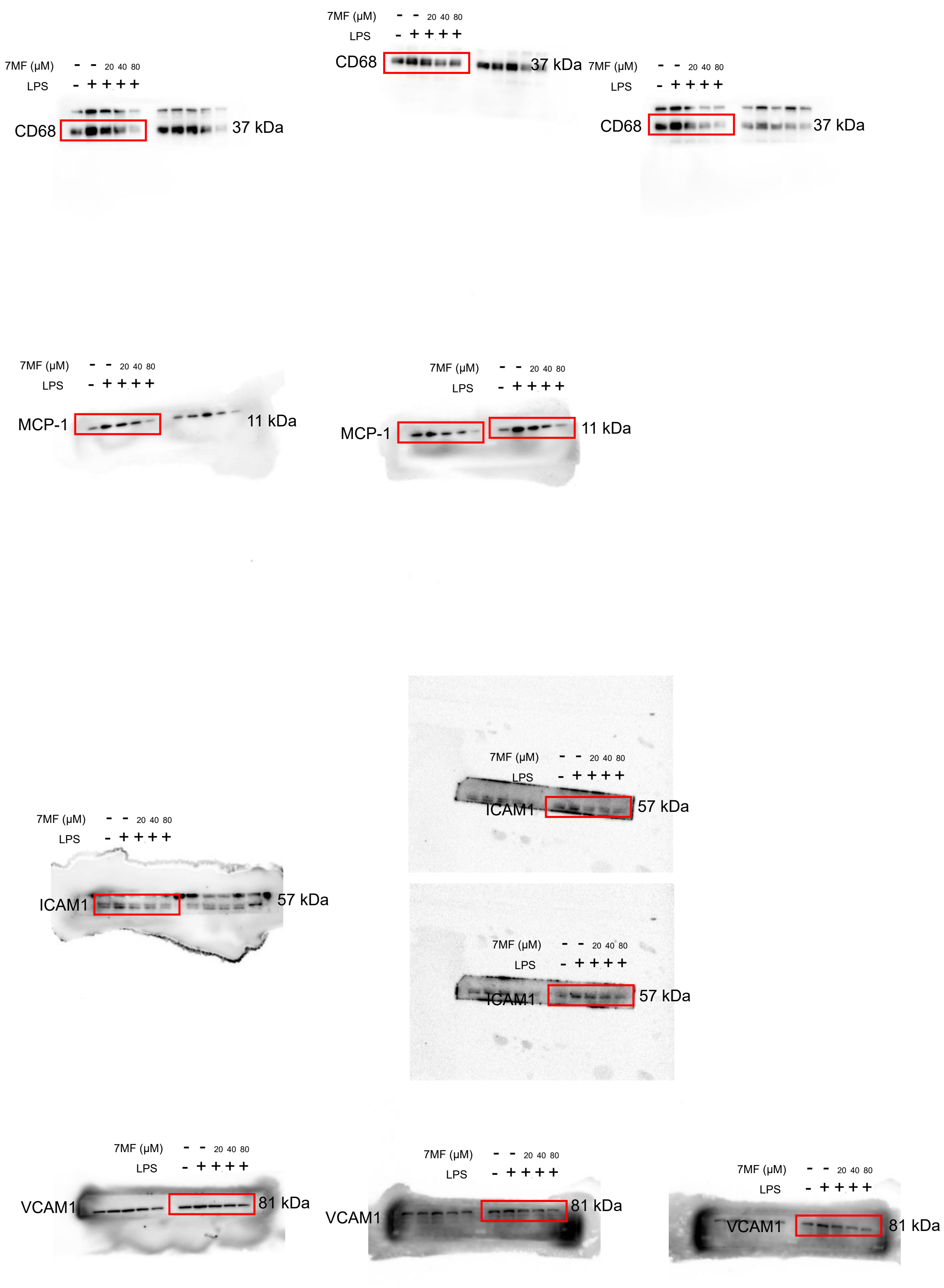
7MF (μM) - - 20 40 80
LPS - + + + +
CD68
37 kDa
MCP-1
11 kDa
ICAM1
57 kDa
VCAM1
81 kDa

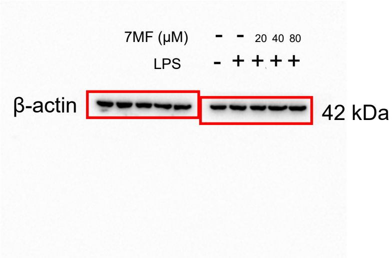
7MF (μM)
- - 20 40 80
LPS
- + + + +
β-actin
42 kDa

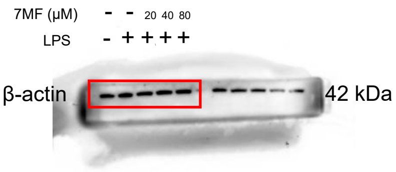
7MF (μM)
- - 20 40 80
LPS
- + + + +
β-actin
42 kDa

# Fig. 3B

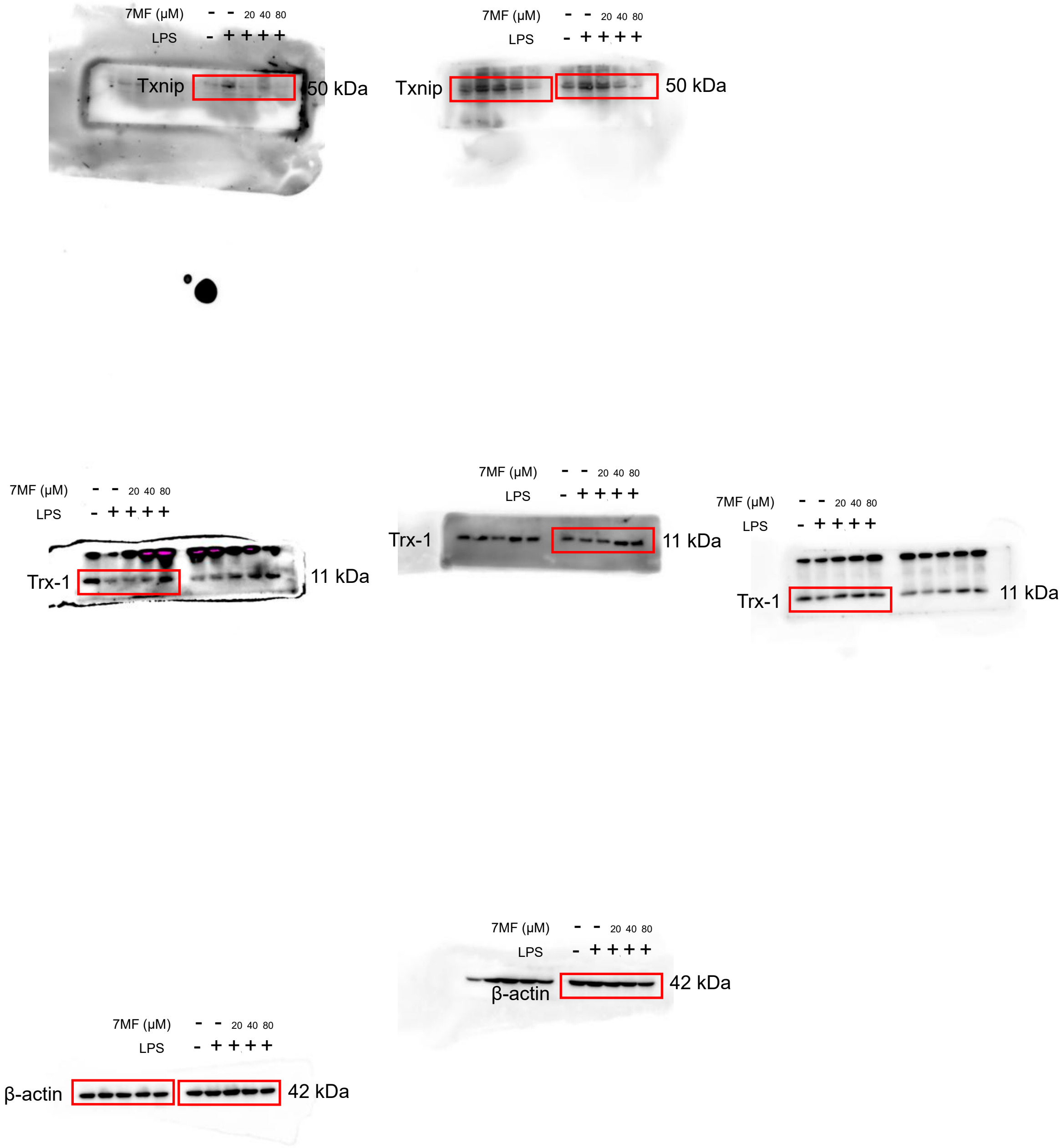

7MF (μM) - - 20 40 80
LPS - + + + +
Txnip
50 kDa
7MF (μM) - - 20 40 80
LPS - + + + +
Txnip
50 kDa
7MF (μM) - - 20 40 80
LPS - + + + +
Trx-1
11 kDa
7MF (μM) - - 20 40 80
LPS - + + + +
Trx-1
11 kDa
7MF (μM) - - 20 40 80
LPS - + + + +
Trx-1
11 kDa
7MF (μM) - - 20 40 80
LPS - + + + +
β-actin
42 kDa
7MF (μM) - - 20 40 80
LPS - + + + +
β-actin
42 kDa

**Fig. 3G**

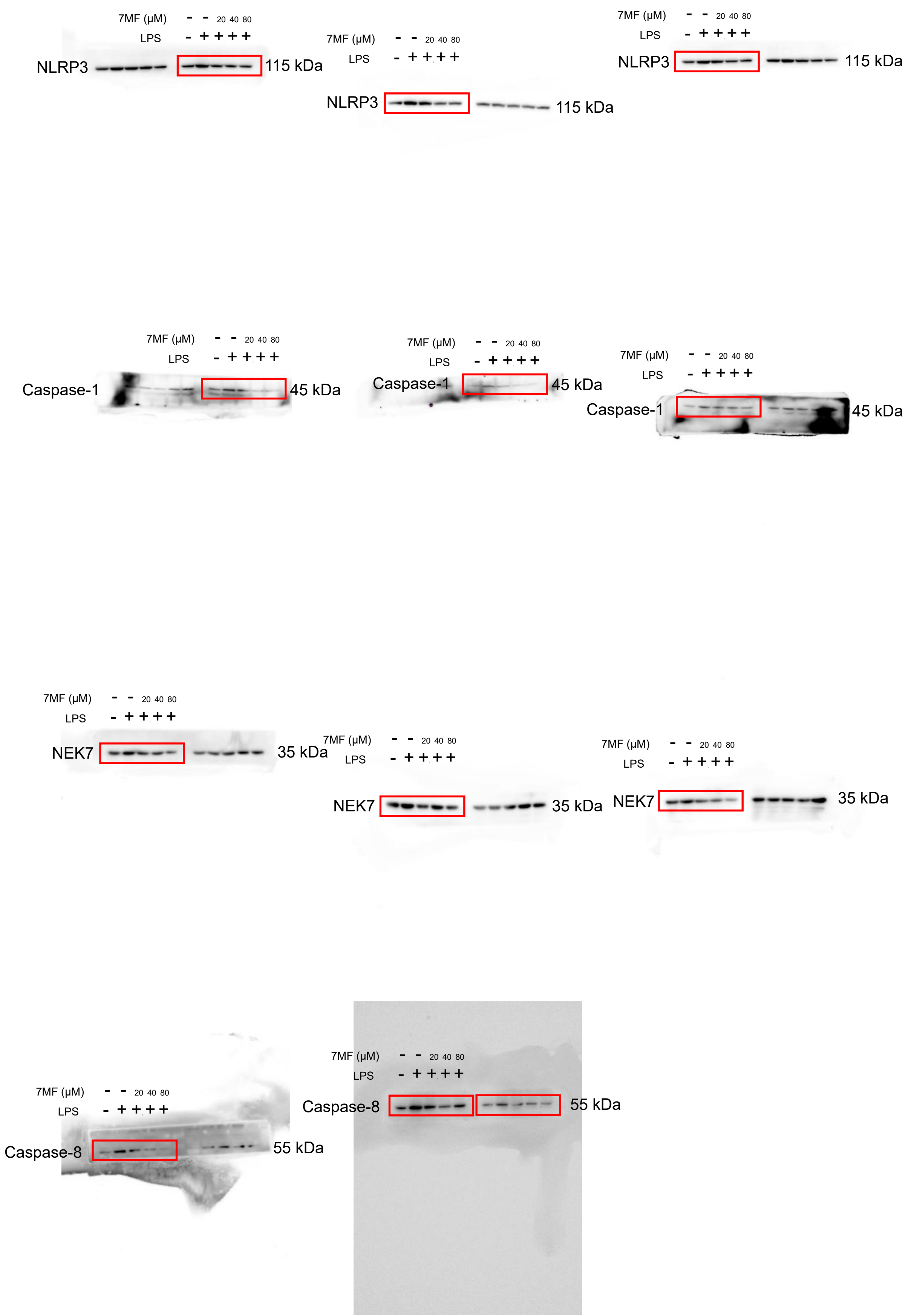
7MF (μM) - - 20 40 80
LPS - + + + +
NLRP3
115 kDa
7MF (μM) - - 20 40 80
LPS - + + + +
NLRP3
115 kDa
7MF (μM) - - 20 40 80
LPS - + + + +
NLRP3
115 kDa
7MF (μM) - - 20 40 80
LPS - + + + +
Caspase-1
45 kDa
7MF (μM) - - 20 40 80
LPS - + + + +
Caspase-1
45 kDa
7MF (μM) - - 20 40 80
LPS - + + + +
Caspase-1
45 kDa
7MF (μM) - - 20 40 80
LPS - + + + +
NEK7
35 kDa
7MF (μM) - - 20 40 80
LPS - + + + +
NEK7
35 kDa
7MF (μM) - - 20 40 80
LPS - + + + +
NEK7
35 kDa
7MF (μM) - - 20 40 80
LPS - + + + +
Caspase-8
55 kDa
7MF (μM) - - 20 40 80
LPS - + + + +
Caspase-8
55 kDa

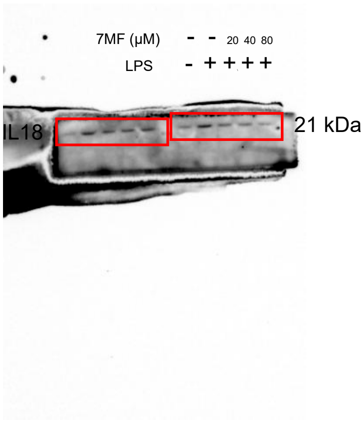
7MF (μM) - - 20 40 80
LPS - + + + +
IL18
21 kDa

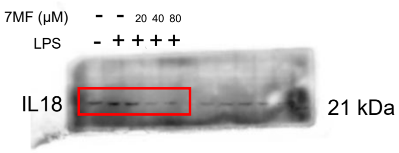
7MF (μM) - - 20 40 80
LPS - + + + +
IL18
21 kDa

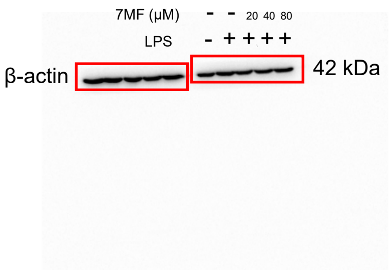
7MF (μM) - - 20 40 80
LPS - + + + +
β-actin
42 kDa

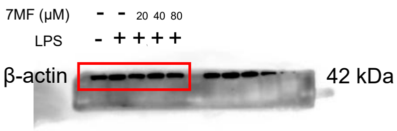
7MF (μM) - - 20 40 80
LPS - + + + +
β-actin
42 kDa

**Fig. 3M**

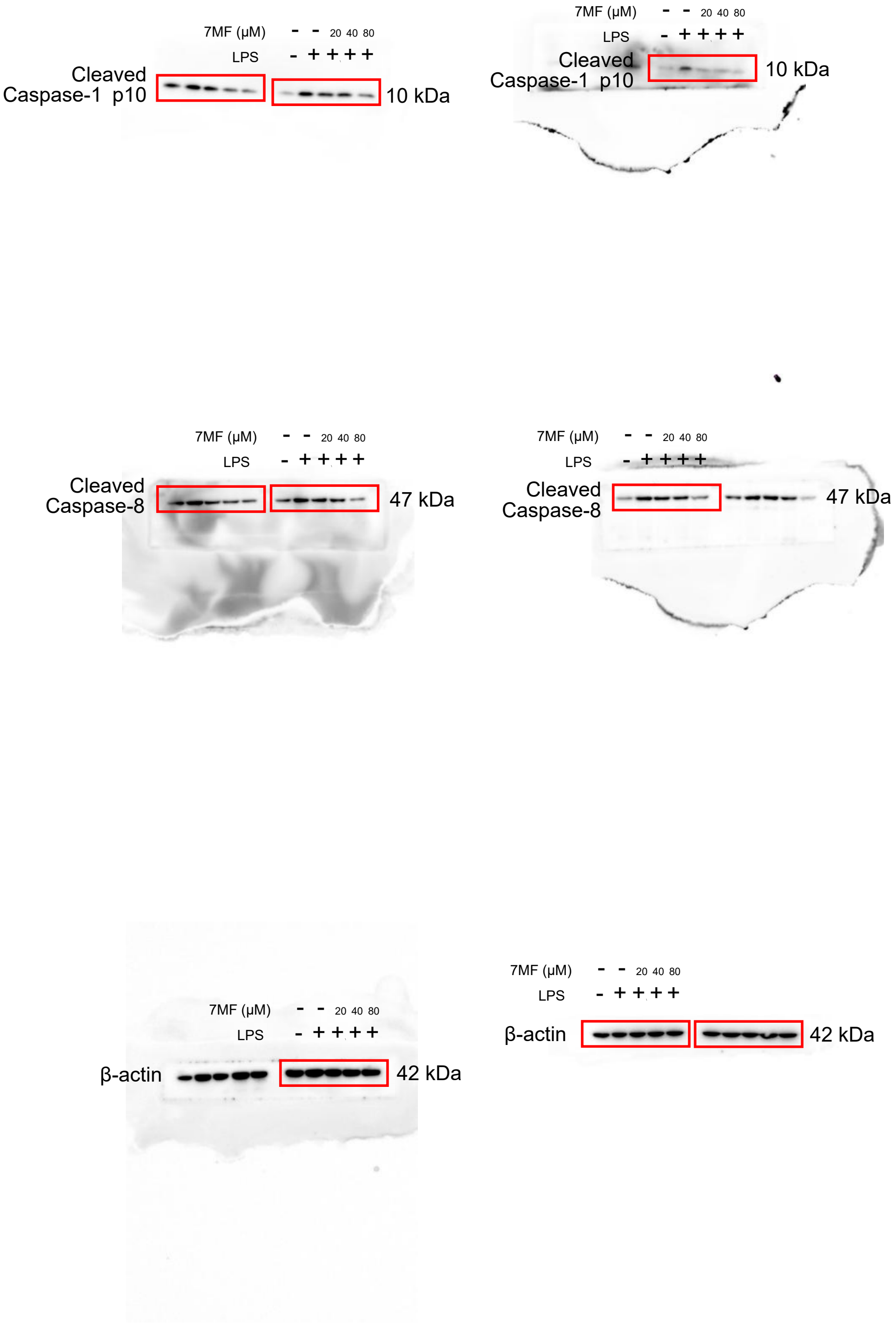
7MF (μM)
- - 20 40 80
LPS
- + + + +
Cleaved Caspase-1 p10
10 kDa
7MF (μM)
- - 20 40 80
LPS
- + + + +
Cleaved Caspase-1 p10
10 kDa
7MF (μM)
- - 20 40 80
LPS
- + + + +
Cleaved Caspase-8
47 kDa
7MF (μM)
- - 20 40 80
LPS
- + + + +
Cleaved Caspase-8
47 kDa
7MF (μM)
- - 20 40 80
LPS
- + + + +
β-actin
42 kDa
7MF (μM)
- - 20 40 80
LPS
- + + + +
β-actin
42 kDa

**Fig. 3P**

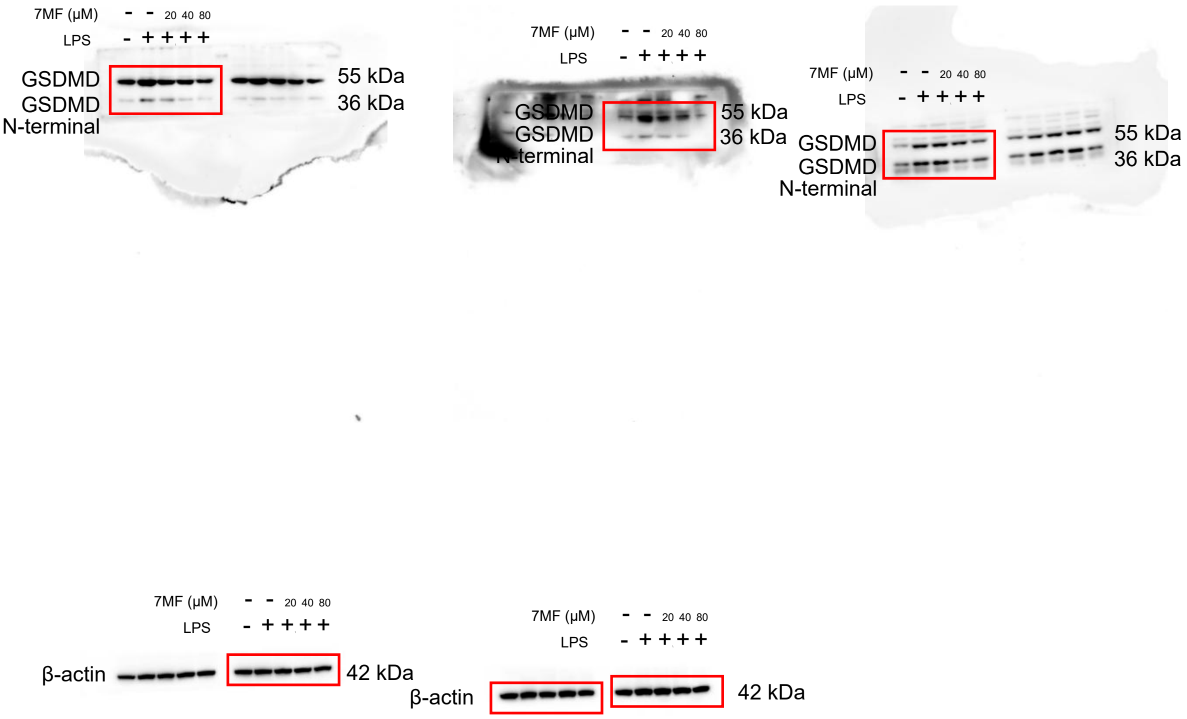
7MF (μM) - - 20 40 80
LPS - + + + +
GSDMD
GSDMD
N-terminal
55 kDa
36 kDa
7MF (μM) - - 20 40 80
LPS - + + + +
GSDMD
GSDMD
N-terminal
55 kDa
36 kDa
7MF (μM) - - 20 40 80
LPS - + + + +
GSDMD
GSDMD
N-terminal
55 kDa
36 kDa
7MF (μM) - - 20 40 80
LPS - + + + +
β-actin
42 kDa
7MF (μM) - - 20 40 80
LPS - + + + +
β-actin
42 kDa

Fig. 3S

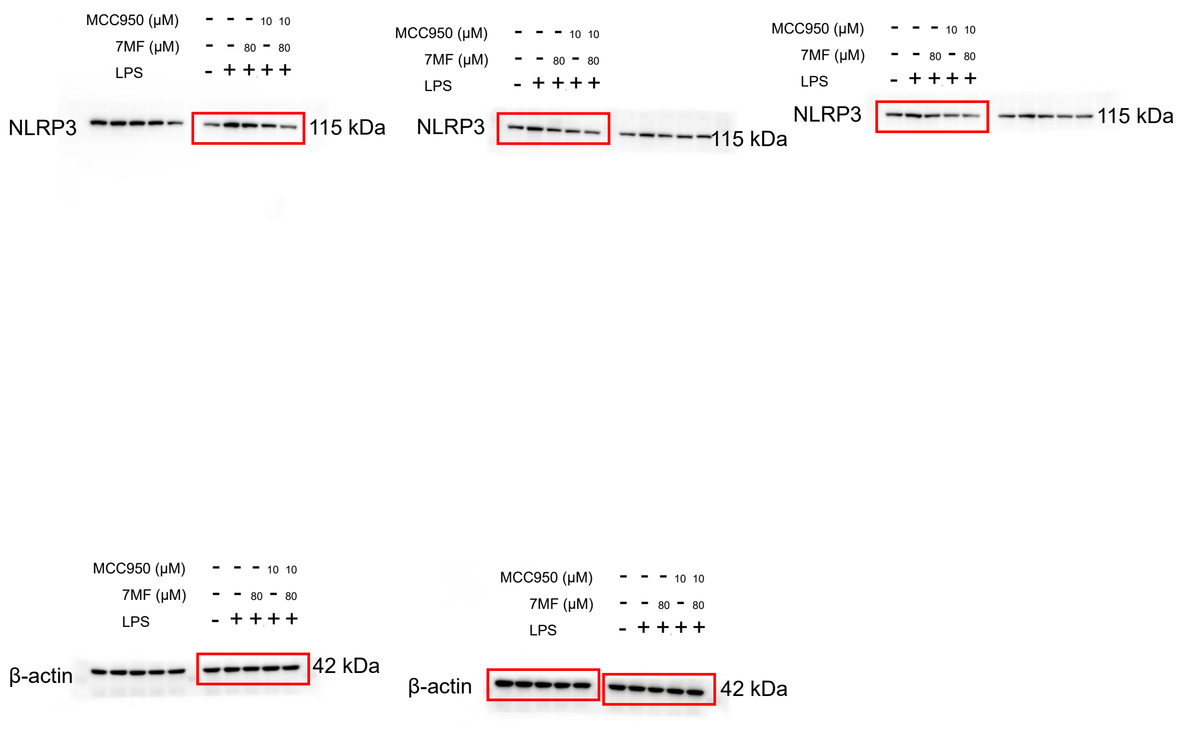

Fig. 5A

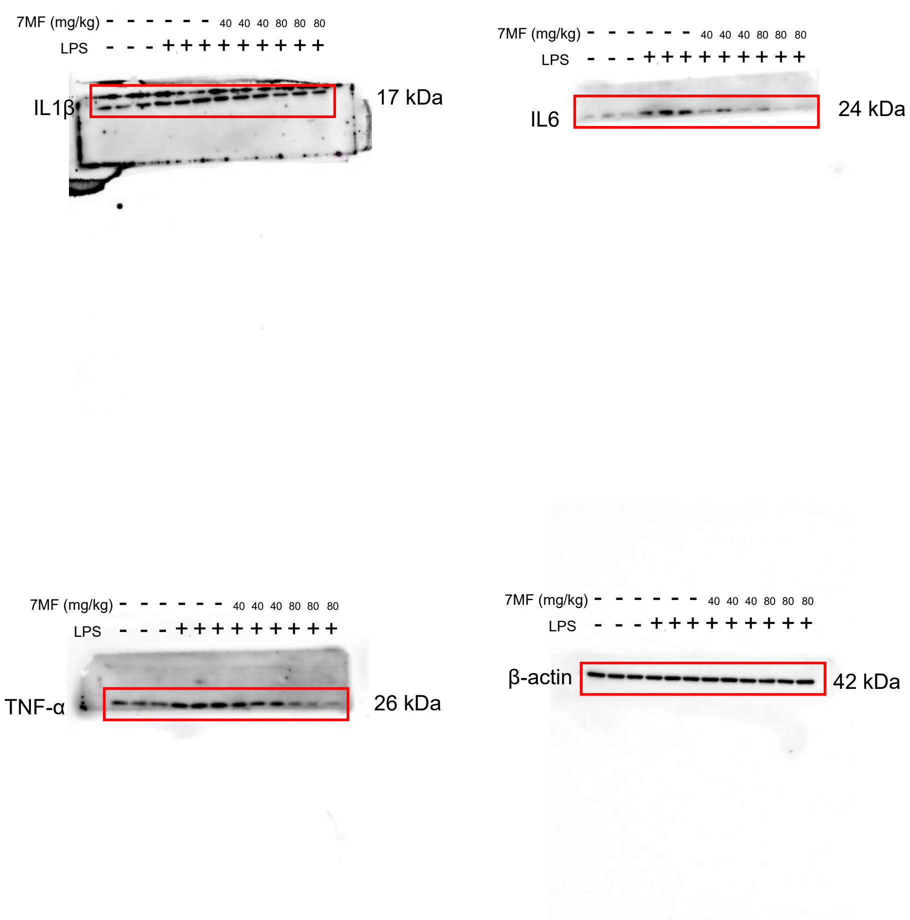
7MF (mg/kg) - - - - - - 40 40 40 80 80 80
LPS - - - + + + + + + + + +
IL1β
17 kDa
7MF (mg/kg) - - - - - - 40 40 40 80 80 80
LPS - - - + + + + + + + + +
IL6
24 kDa
7MF (mg/kg) - - - - - - 40 40 40 80 80 80
LPS - - - + + + + + + + + +
TNF-α
26 kDa
7MF (mg/kg) - - - - - - 40 40 40 80 80 80
LPS - - - + + + + + + + + +
β-actin
42 kDa

**Fig. 5E**

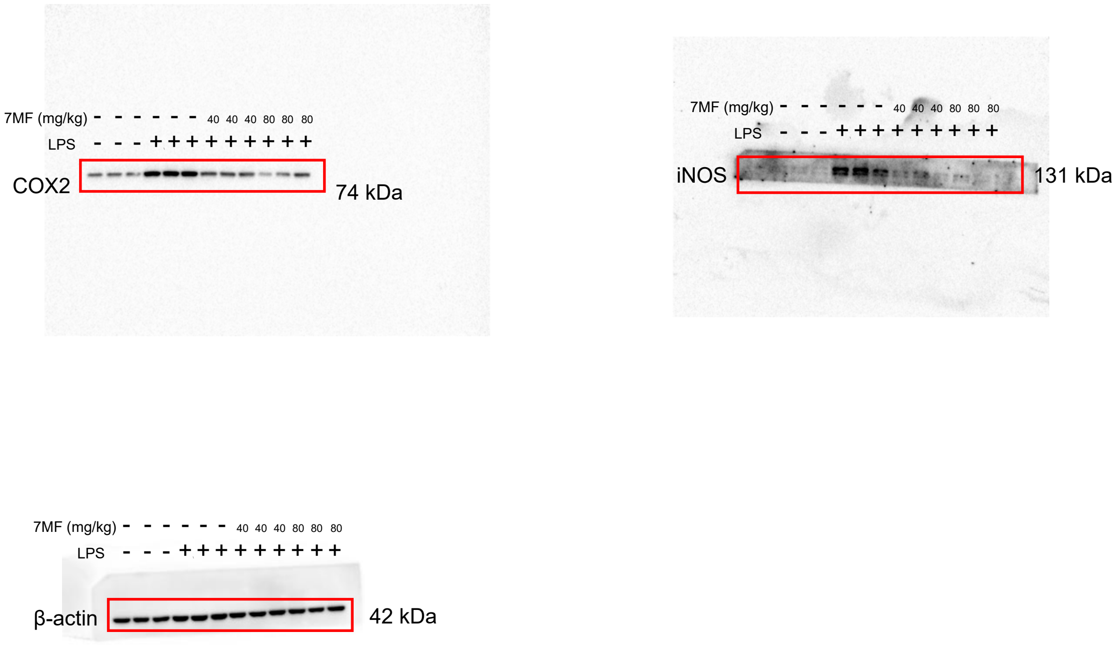
7MF (mg/kg) - - - - - - 40 40 40 80 80 80
LPS - - - + + + + + + + + +
COX2
74 kDa
7MF (mg/kg) - - - - - - 40 40 40 80 80 80
LPS - - - + + + + + + + + +
iNOS
131 kDa
7MF (mg/kg) - - - - - - 40 40 40 80 80 80
LPS - - - + + + + + + + + +
β-actin
42 kDa

**Fig. 6B**

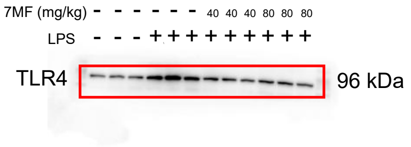
7MF (mg/kg) - - - - - - 40 40 40 80 80 80
LPS - - - + + + + + + + + +
TLR4
96 kDa

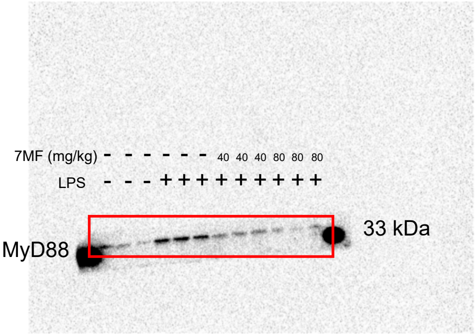
7MF (mg/kg) - - - - - - 40 40 40 80 80 80
LPS - - - + + + + + + + + +
MyD88
33 kDa

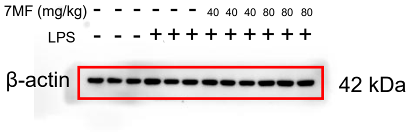
7MF (mg/kg) - - - - - - 40 40 40 80 80 80
LPS - - - + + + + + + + + +
β-actin
42 kDa

**Fig. 6E**

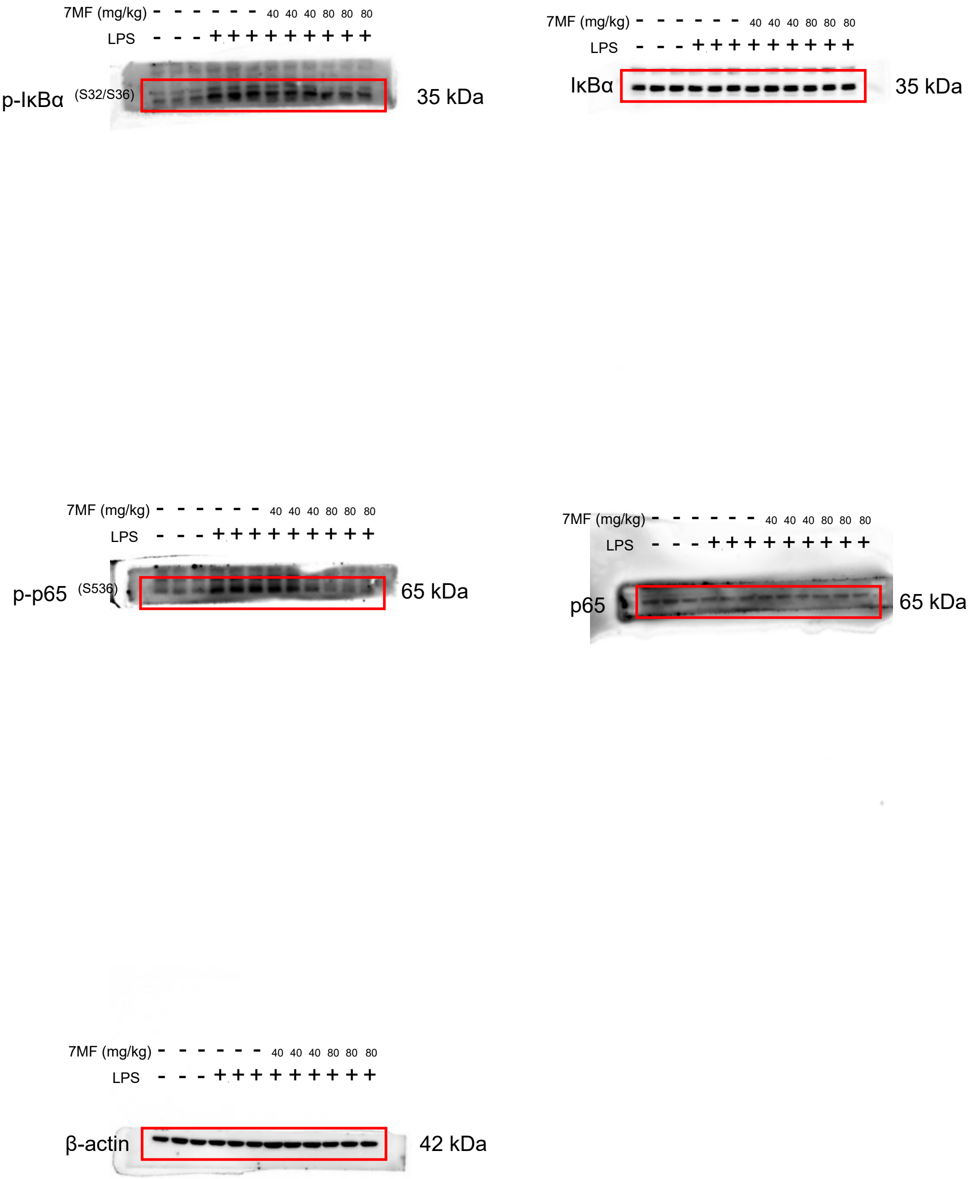

7MF (mg/kg) - - - - - - 40 40 40 80 80 80
LPS - - - + + + + + + + + +
p-IκBα (S32/S36)
35 kDa
7MF (mg/kg) - - - - - - 40 40 40 80 80 80
LPS - - - + + + + + + + + +
IκBα
35 kDa
7MF (mg/kg) - - - - - - 40 40 40 80 80 80
LPS - - - + + + + + + + + +
p-p65 (S536)
65 kDa
7MF (mg/kg) - - - - - - 40 40 40 80 80 80
LPS - - - + + + + + + + + +
p65
65 kDa
7MF (mg/kg) - - - - - - 40 40 40 80 80 80
LPS - - - + + + + + + + + +
β-actin
42 kDa

**Fig. 6H**

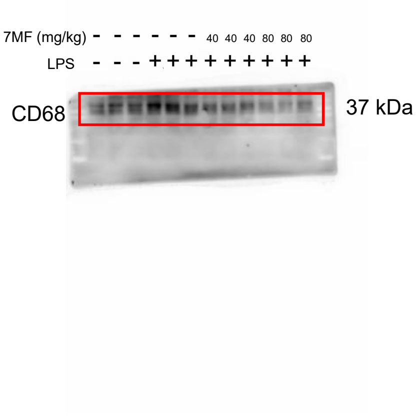
7MF (mg/kg) - - - - - - 40 40 40 80 80 80
LPS - - - + + + + + + + + +
CD68
37 kDa

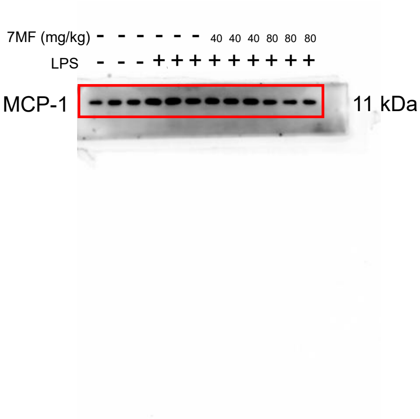
7MF (mg/kg) - - - - - - 40 40 40 80 80 80
LPS - - - + + + + + + + + +
MCP-1
11 kDa

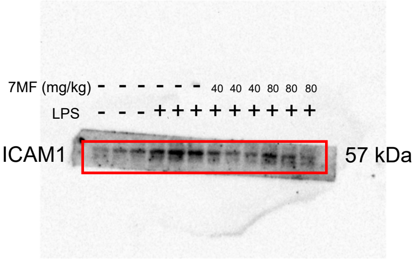
7MF (mg/kg) - - - - - - 40 40 40 80 80 80
LPS - - - + + + + + + + + +
ICAM1
57 kDa

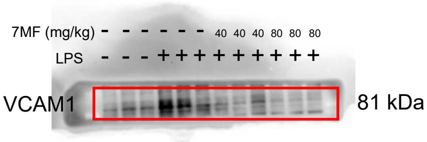
7MF (mg/kg) - - - - - - 40 40 40 80 80 80
LPS - - - + + + + + + + + +
VCAM1
81 kDa

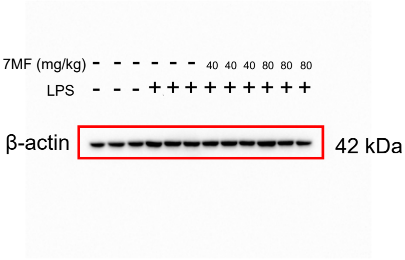
7MF (mg/kg) - - - - - - 40 40 40 80 80 80
LPS - - - + + + + + + + + +
β-actin
42 kDa

**Fig. 7A**

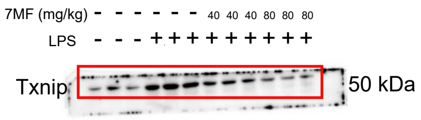

7MF (mg/kg) - - - - - - 40 40 40 80 80 80
LPS - - - + + + + + + + + +
Txnip
50 kDa


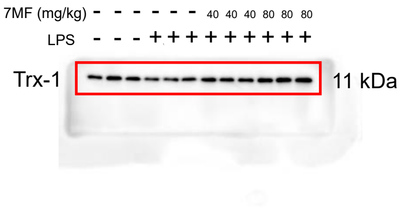

7MF (mg/kg) - - - - - - 40 40 40 80 80 80
LPS - - - + + + + + + + + +
Trx-1
11 kDa


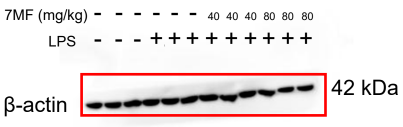

7MF (mg/kg) - - - - - - 40 40 40 80 80 80
LPS - - - + + + + + + + + +
β-actin
42 kDa

**Fig. 7C**

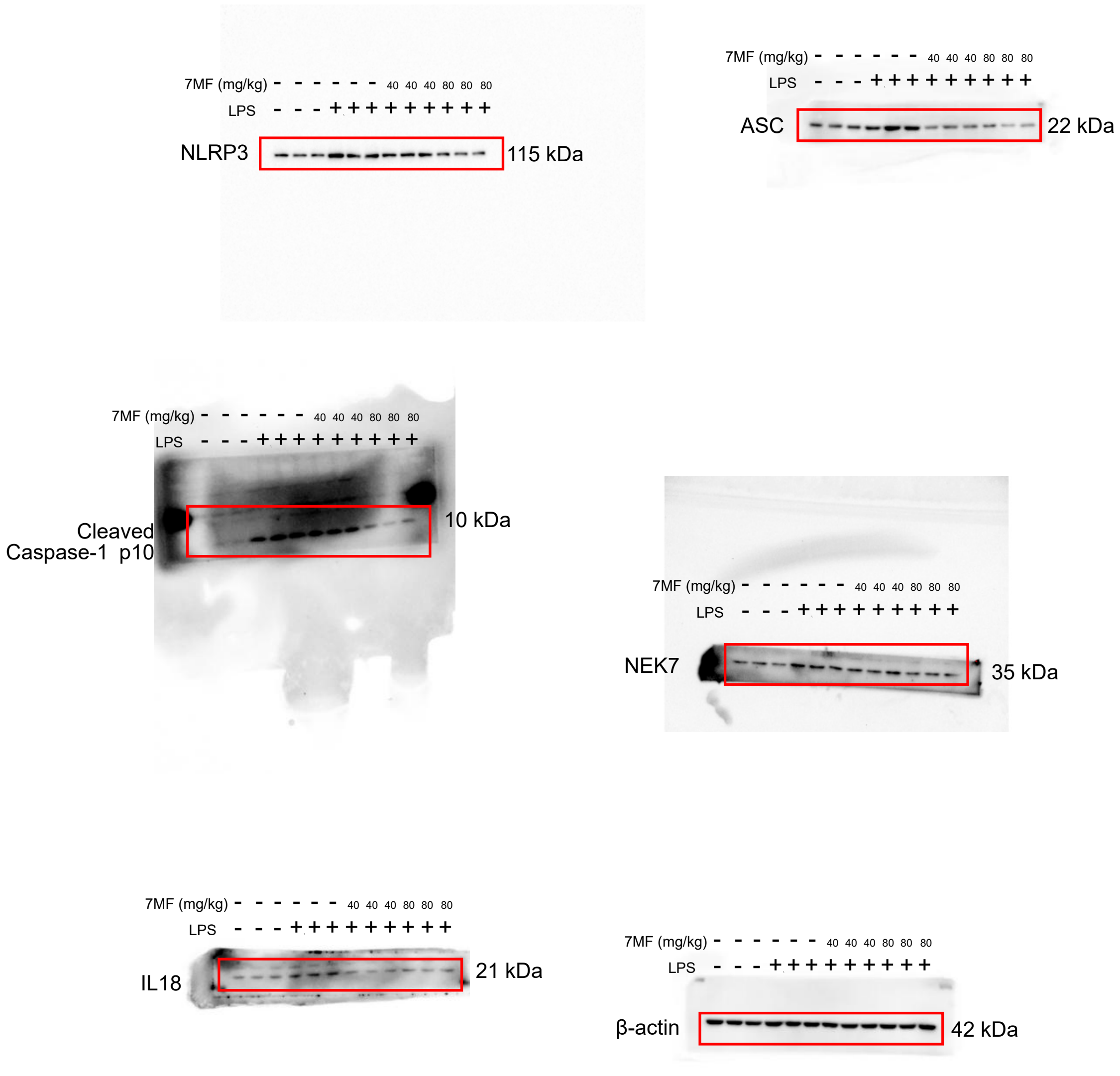
7MF (mg/kg) - - - - - - 40 40 40 80 80 80
LPS - - - + + + + + + + + +
NLRP3
115 kDa
7MF (mg/kg) - - - - - - 40 40 40 80 80 80
LPS - - - + + + + + + + + +
ASC
22 kDa
7MF (mg/kg) - - - - - - 40 40 40 80 80 80
LPS - - - + + + + + + + + +
Cleaved Caspase-1 p10
10 kDa
7MF (mg/kg) - - - - - - 40 40 40 80 80 80
LPS - - - + + + + + + + + +
NEK7
35 kDa
7MF (mg/kg) - - - - - - 40 40 40 80 80 80
LPS - - - + + + + + + + + +
IL18
21 kDa
7MF (mg/kg) - - - - - - 40 40 40 80 80 80
LPS - - - + + + + + + + + +
β-actin
42 kDa

**Fig. 7J**

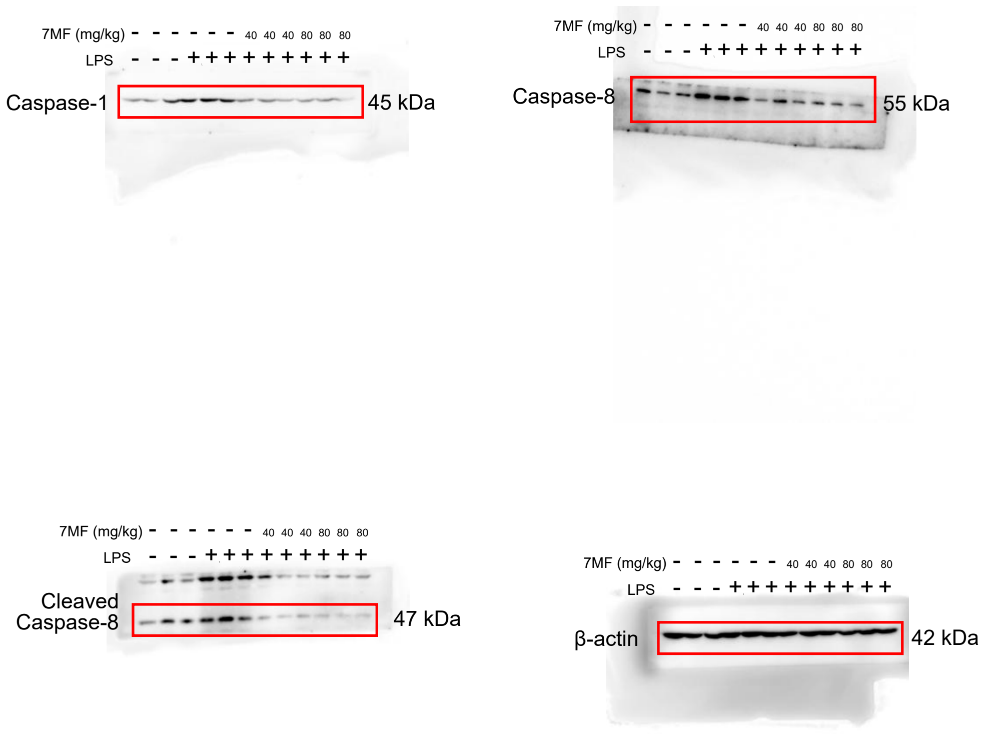

7MF (mg/kg) - - - - - - 40 40 40 80 80 80
LPS - - - + + + + + + + + +
Caspase-1
45 kDa
7MF (mg/kg) - - - - - - 40 40 40 80 80 80
LPS - - - + + + + + + + + +
Caspase-8
55 kDa
7MF (mg/kg) - - - - - - 40 40 40 80 80 80
LPS - - - + + + + + + + + +
Cleaved Caspase-8
47 kDa
7MF (mg/kg) - - - - - - 40 40 40 80 80 80
LPS - - - + + + + + + + + +
β-actin
42 kDa

**Fig. 7N**

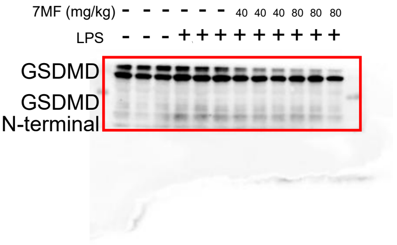
7MF (mg/kg) - - - - - - 40 40 40 80 80 80
LPS - - - + + + + + + + + +
GSDMD
GSDMD
N-terminal

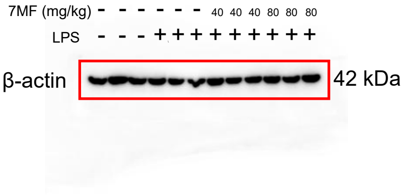
7MF (mg/kg) - - - - - - 40 40 40 80 80 80
LPS - - - + + + + + + + + +
β-actin
42 kDa

**Tissue sections images**

**Fig. 4B**

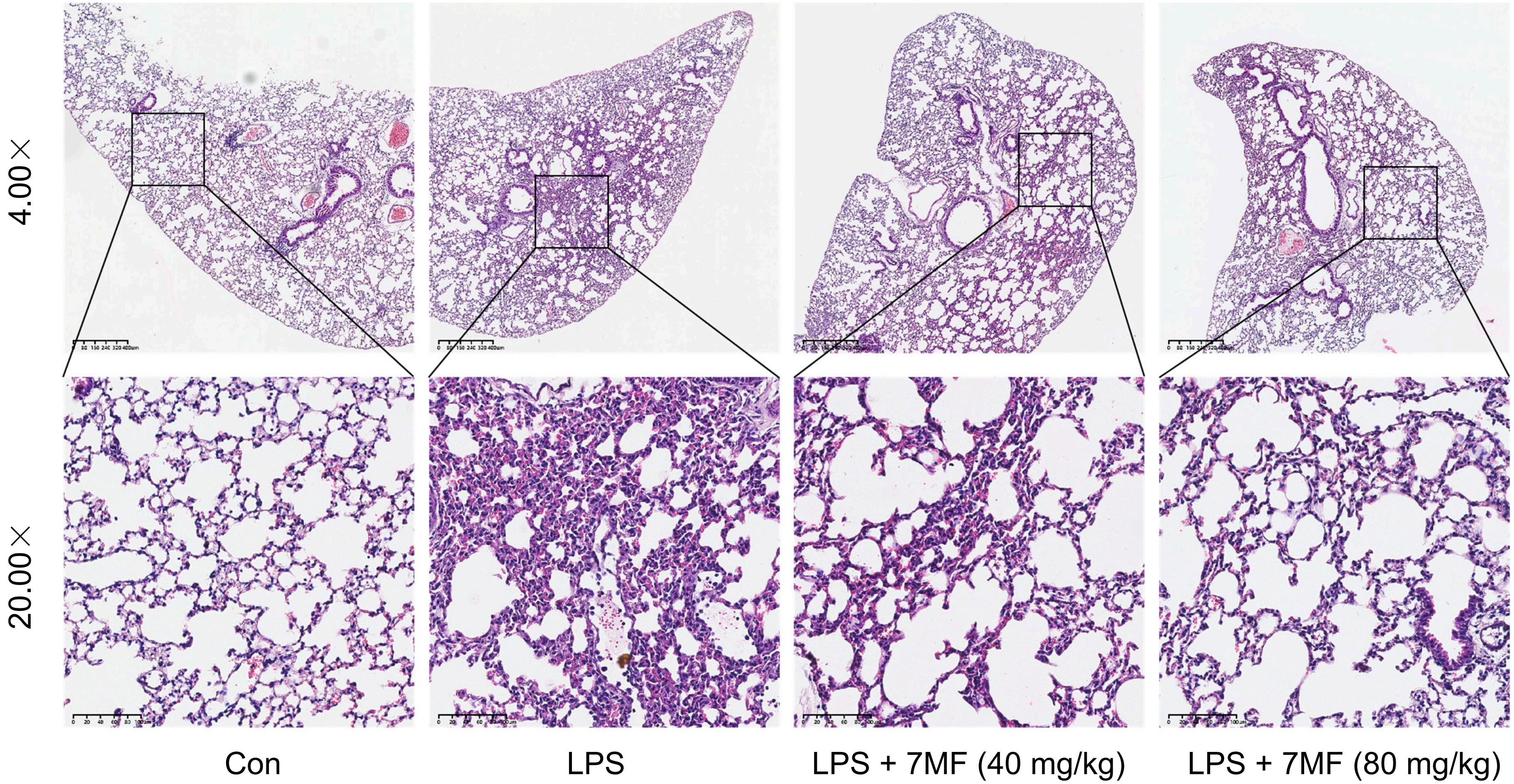

**Fig. 5D**

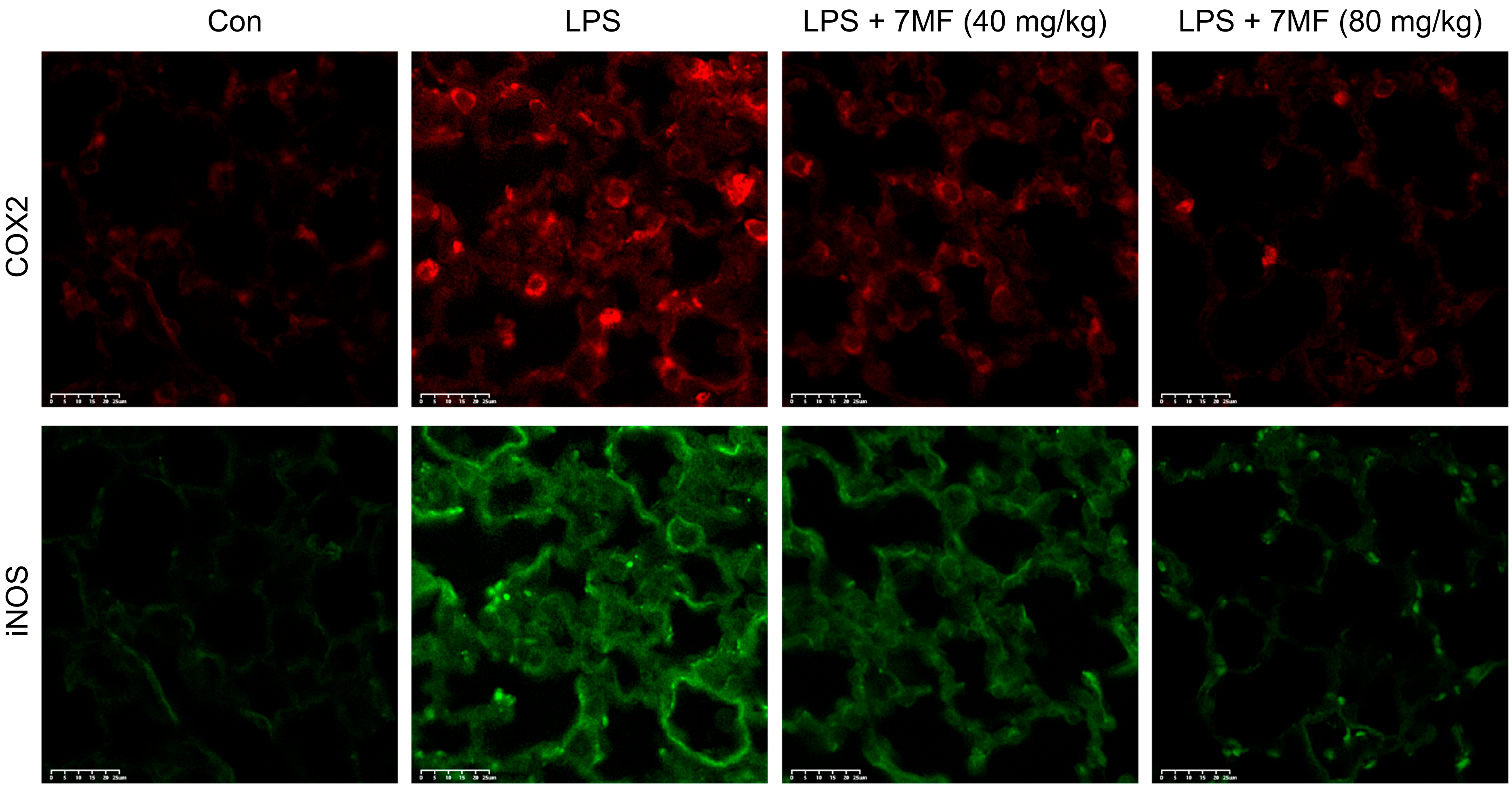

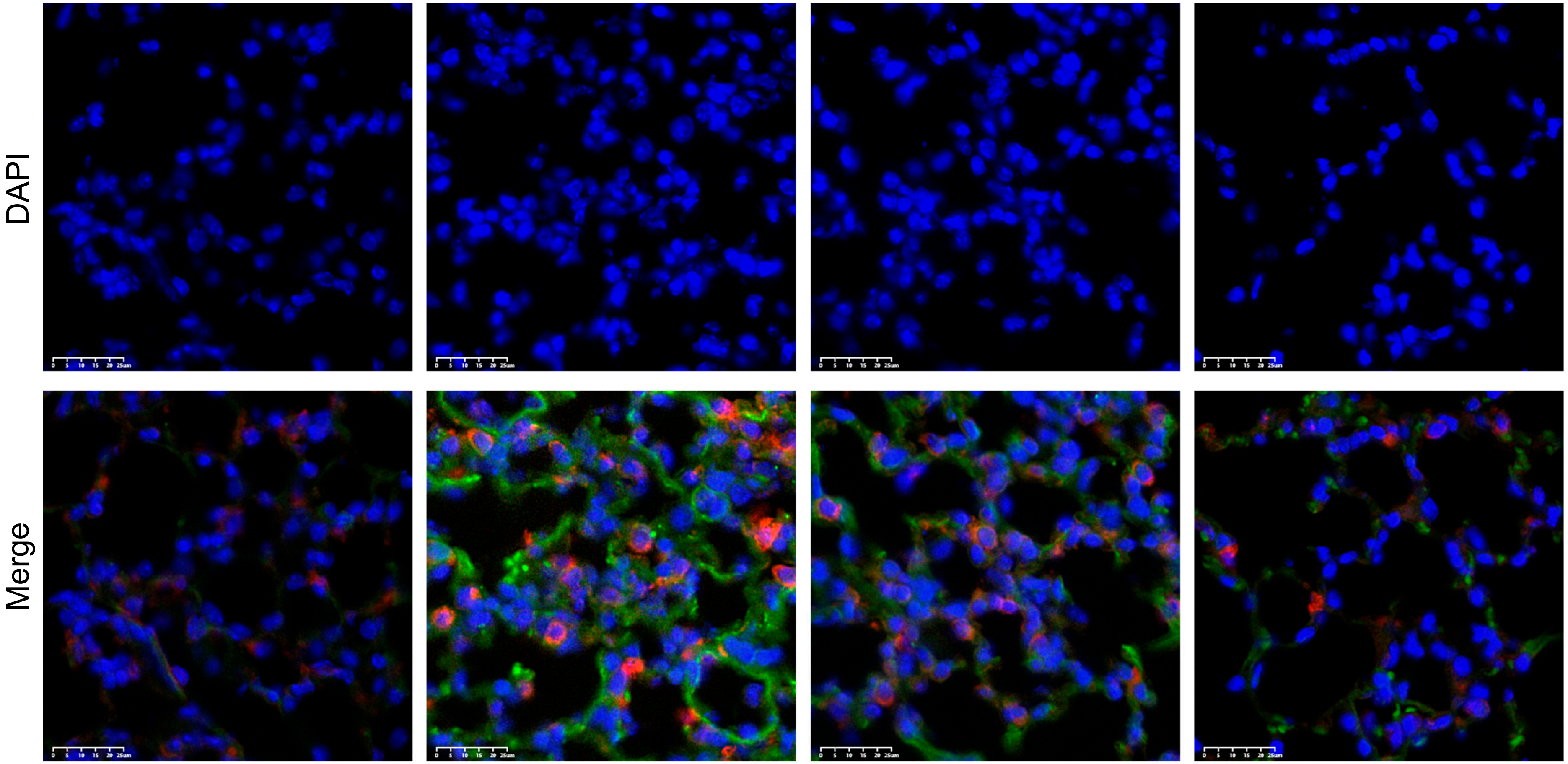
DAPI
Merge

**Fig. 6A**

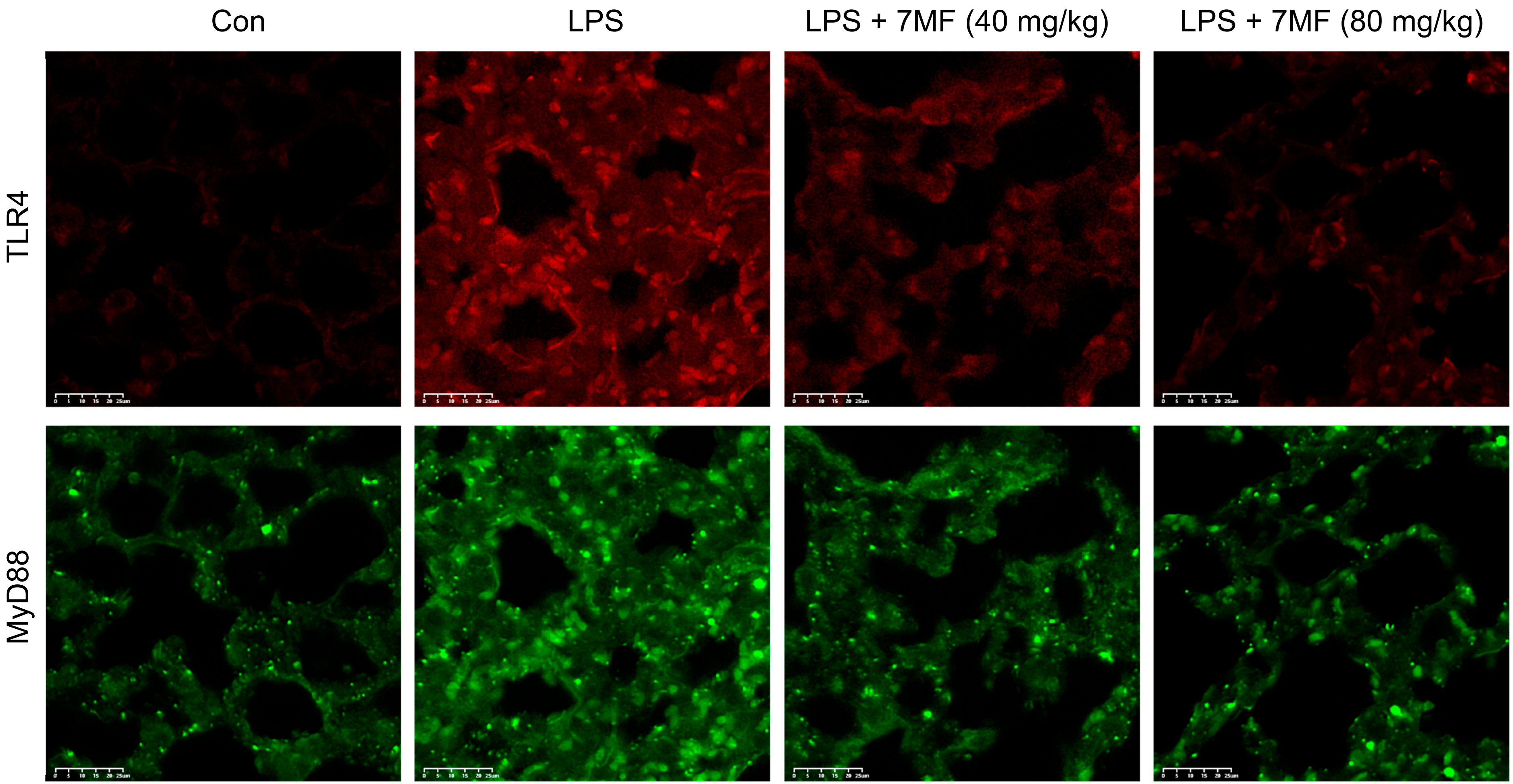

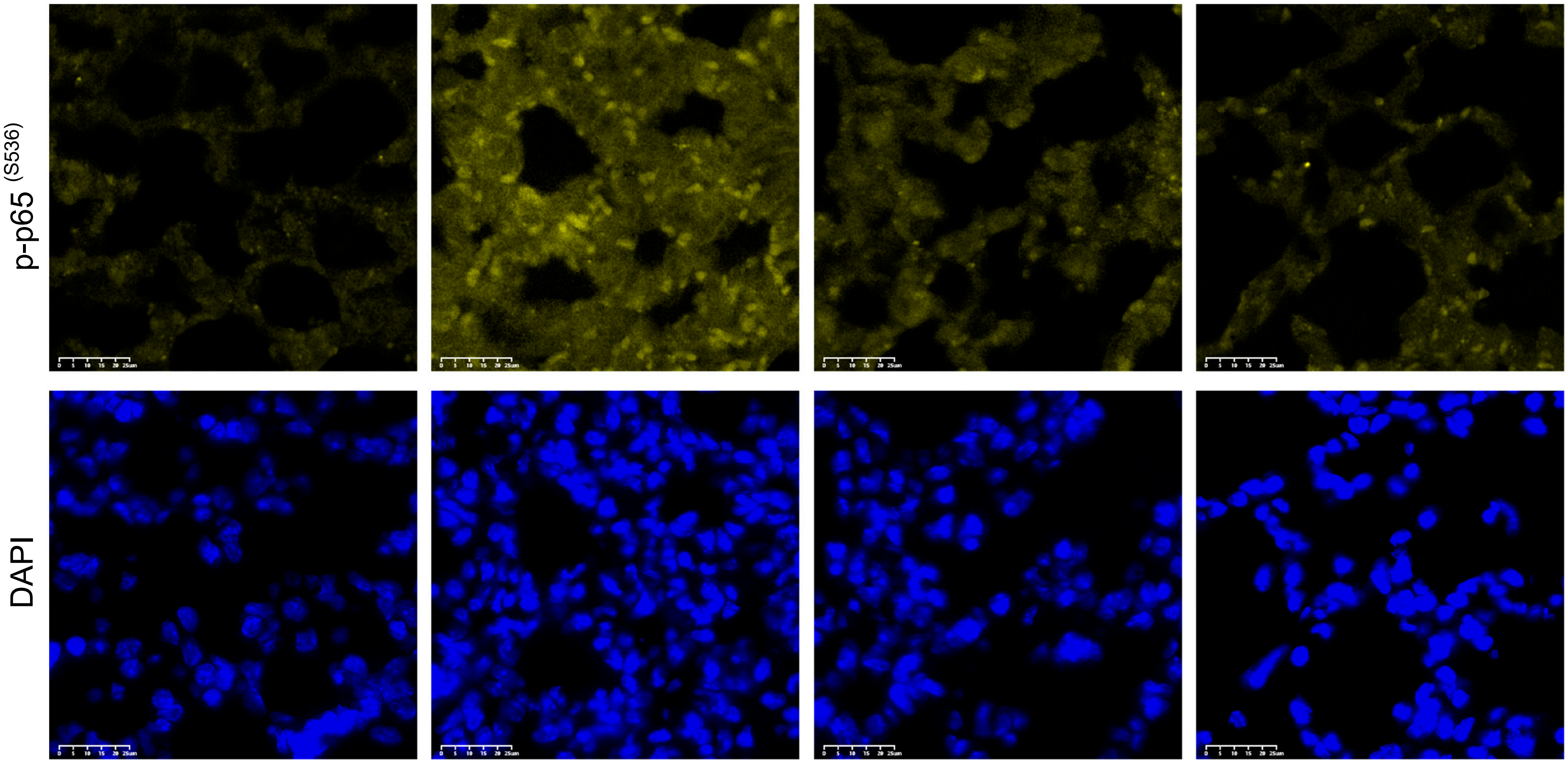
p-p65 (S536)
DAPI
0 5 10 15 20 25um

Merge

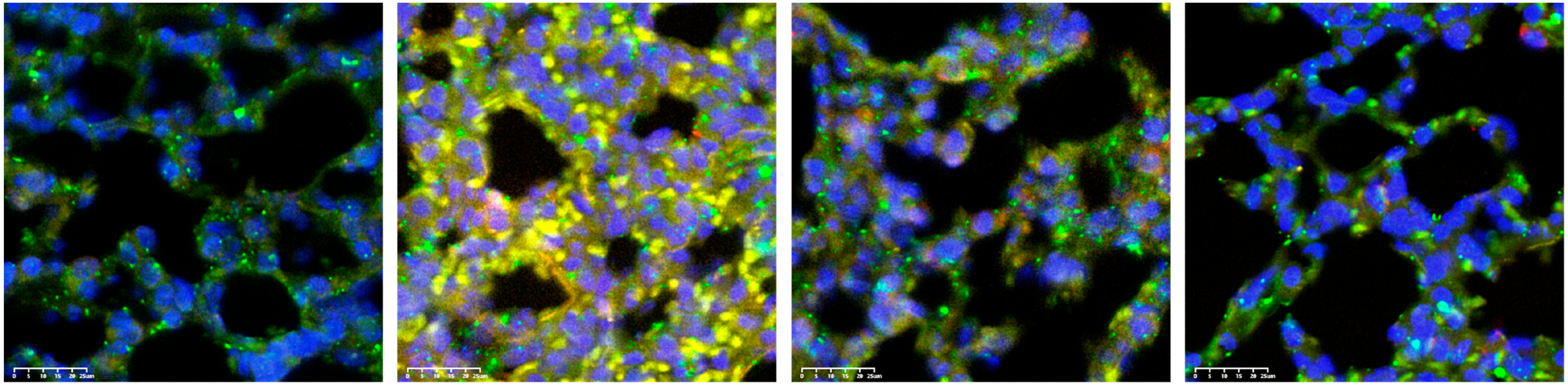

**Fig. 7I**

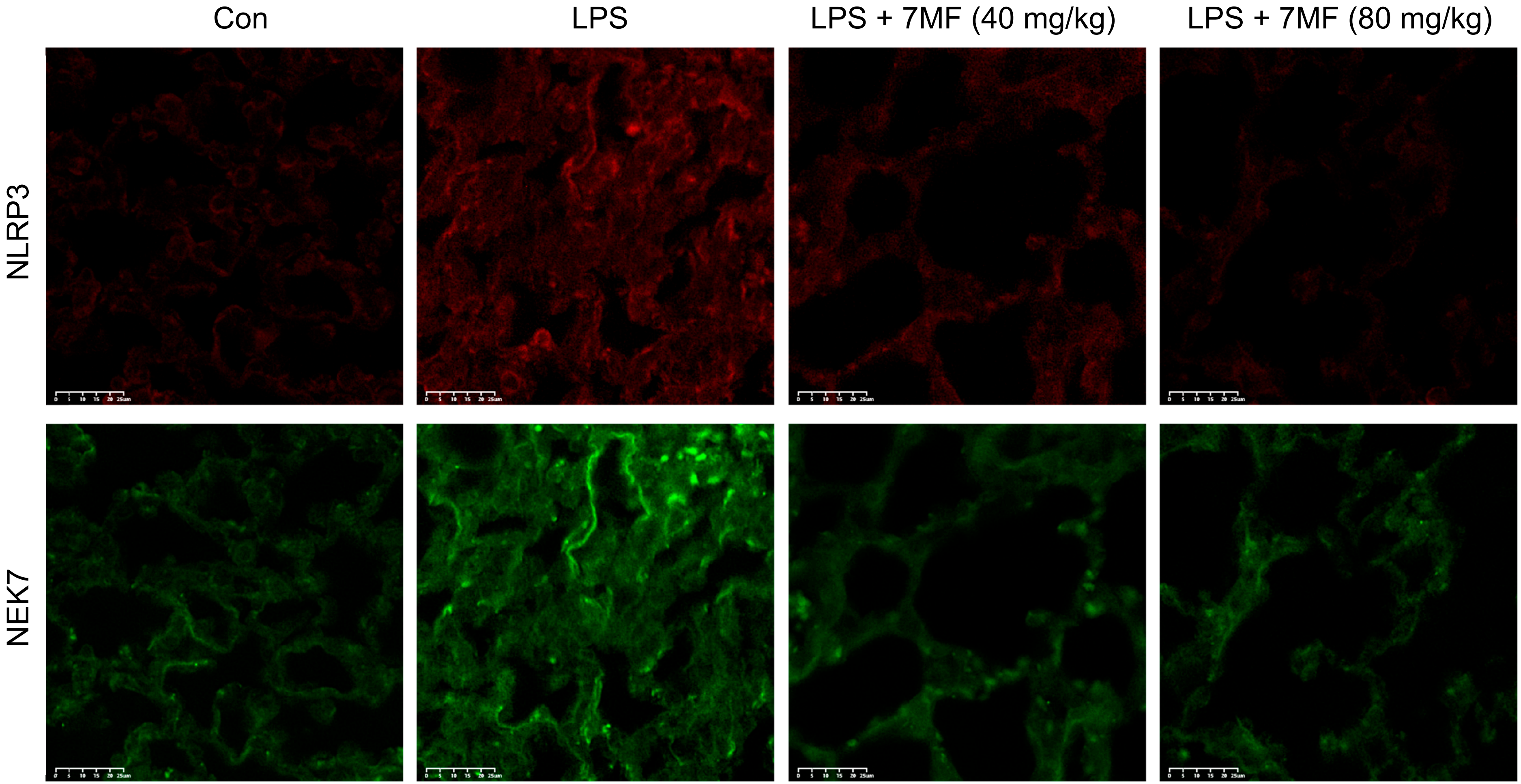

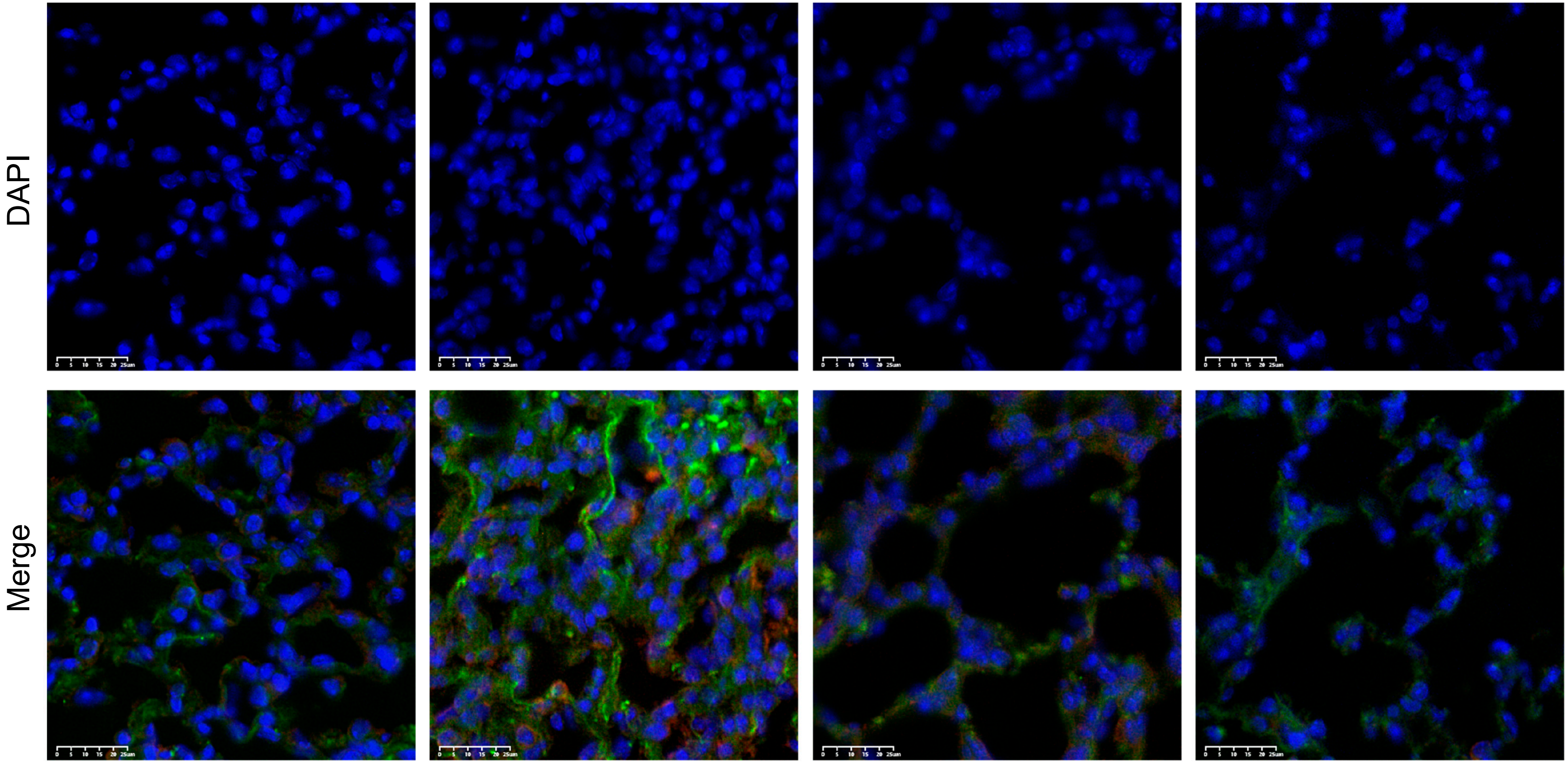
DAPI
Merge
